# Exploring Anti-Prion Glyco-Based and Aromatic Scaffolds: A Chemical Strategy for the Quality of Life

**DOI:** 10.3390/molecules22060864

**Published:** 2017-05-24

**Authors:** María Teresa Blázquez-Sánchez, Ana M. de Matos, Amélia P. Rauter

**Affiliations:** Centro de Química e Bioquímica, Faculdade de Ciências, Universidade de Lisboa, Ed C8, Piso 5, Campo Grande, 1749-016 Lisboa, Portugal; mblazquez@fc.ul.pt (M.T.B.-S.); amamatos@fc.ul.pt (A.M.d.M.)

**Keywords:** prion, protein aggregation, amyloid, anti-prion compounds, structure optimization

## Abstract

Prion diseases are fatal neurodegenerative disorders caused by protein misfolding and aggregation, affecting the brain progressively and consequently the quality of life. Alzheimer’s is also a protein misfolding disease, causing dementia in over 40 million people worldwide. There are no therapeutics able to cure these diseases. Cellular prion protein is a high-affinity binding partner of amyloid β (Aβ) oligomers, the most toxic species in Alzheimer’s pathology. These findings motivate the development of new chemicals for a better understanding of the events involved. Disease control is far from being reached by the presently known therapeutics. In this review we describe the synthesis and mode of action of molecular entities with intervention in prion diseases’ biological processes and, if known, their role in Alzheimer’s. A diversity of structures is covered, based on glycans, steroids and terpenes, heterocycles, polyphenols, most of them embodying aromatics and a structural complexity. These molecules may be regarded as chemical tools to foster the understanding of the complex mechanisms involved, and to encourage the scientific community towards further developments for the cure of these devastating diseases.

## 1. Introduction

Prion diseases, also called transmissible spongiform encephalopathies (TSEs), are infectious and fatal neurodegenerative diseases. They affect the brain and the nervous system, causing deterioration of mental and physical abilities, amyloid plaque formation, neuronal death, and production of holes in the cortex that appears, when examined under a microscope, as a sponge. The clinical signs in humans may include memory problems and personality changes, depression, lack of coordination, involuntary movements or insomnia. In the later stages of the disease, dementia and loss of ability to move or speak are common. Prion diseases are the single conformational diseases transmissible, either experimentally or by natural routes, mainly by ingestion. Prions have an extraordinary resistance to conventional sterilization procedures and are capable to bind to metal and plastic surfaces without losing infectivity. They are caused by an abnormal conformational isoform (PrP^Sc^) of the host-encoded *cellular* prion protein (PrP^C^) [1]. Full-length PrP^C^ is a 253 amino acid-long glycoprotein ubiquitously expressed in all mammals and anchored to the external cell membrane by a glycosylphosphatidylinositol (GPI) moiety [2]. Although there is evidence on its relevant physiological role as a cellular signalling molecule [3,4,5], once in the presence of infectious PrP^Sc^ seeds, PrP^C^ units are remodelled to reproduce the conformation of the misfolded isoform. Newly formed PrP^Sc^ molecules are then incorporated into prion amyloid aggregates that tend to accumulate in neurons, astroglia and microglia, eventually leading to synaptic damage and vacuolar neuropathology characteristic of TSEs [6]. The exact mechanism by which PrP^Sc^ aggregates lead to neurotoxicity is, nevertheless, poorly understood.

Human TSEs are rare and include familial Creutzfeld-Jacob disease (CJD) caused by polymorphisms in the *PRNP* gene giving rise to PrP^Sc^, sporadic CJD, as well as the variant CJD triggered by the consumption of bovine PrP^Sc^-contaminated food [1,7]. Attempts to develop small organic compounds targeting either PrP^C^, PrP^Sc^, or the conversion from one conformer to another have been carried out in the past two decades, but even though a few orally available compounds have been shown to significantly enhance the survival of mice infected with mouse PrP^Sc^, the transposition of such effects to human PrP^Sc^ has been challenging and led to rather disappointing results so far. The reasons for this include undesired toxicity and metabolic profile or, in some cases, reduced compound blood brain barrier permeability [8]. Yet, the existence of various phenotypic TSE variants caused by several different PrP^Sc^ strains is possibly the major factor contributing to these consecutive failures in the search for effective therapeutic approaches. Each PrP^Sc^ strain leads to a distinct disease incubation period, and each one is associated with a particular pattern of prion distribution in the brain [1,9,10] and, indeed, it has been shown that one particular molecule may exert distinct effects against different prion strains [11].

Despite so many difficulties, the urgent need for efficacious treatments against prion diseases keeps the scientific community motivated to pursuing the discovery of new anti-prion molecules up until today. In this perspective, we herein provide an overview of the latest reports focusing on the development of molecules with anti-prion activity and potential against TSEs. From sugar-based compounds to aromatic and heteroaromatic compounds, this review presents also the synthesis and mode of action of the most promising molecular entities described in the literature, ultimately aiming to stimulate the development of innovative therapies against prion-mediated diseases.

## 2. Synthesis and Mode of Action of Glyco-Based and Aromatic Scaffolds with anti-Prion Effects

### 2.1. Glyco-Based Molecular Entities

**Figure 1:**
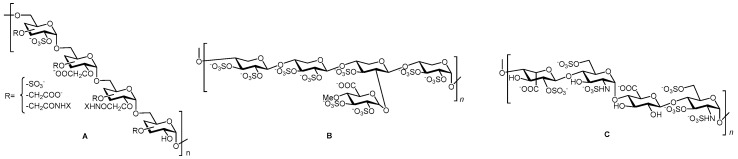
(**A**): Heparan mimetic representative fragment, abbreviated as HMCSX where n: molecular length, S: degree of sulfate substitution, C: carboxylate substitution and X: hydrophobic group substituent; (**B**): Pentosan polysulfate representative fragment; (**C**): Heparan sulfate/heparin representative fragment.

Sulfated polysaccharides, as the family of heparan mimetics (HMs) and pentose polysulfate (PPS) are among the most active drugs tested in experimental models of prion disease (Figure 1, structures A–C). Heparan sulfate (HS) was found on the amyloid plaques in TSEs [12] and reported as an essential part of the cellular receptor used for prion uptake and as a crucial factor for cell infection [13]. It is known that sulfation degree and patterns of these polysaccharide structures govern the HS-protein interactions [14] and are related to their anti-prion acitivity. Other endogenous polysaccharides including heparin, chondroitin sulfate (CS) and dermatan sulfate (DS), have also shown to bind prions [15,16], and to inhibit the neurotoxicity of amyloid fibrils [17].

Based on these findings, Ouidja and co-workers [18] reported a library of HMs of different molecular sizes, containing various sulfation and carboxylation levels, and substituted, or not, by different hydrophobic cores with a general formula HMCSX. (Figure 1, structure A). Studies on their capacity to inhibit the accumulation of the abnormal protease-resistant prion conformation (PrP^res^) in chronically infected cells (ScGT1-7) and on their PrP^rec^ binding ability showed that an optimal size and sulfation degree were needed for optimum activity. The incorporation of hydrophobic moieties increased compound efficacy while the presence of carboxymethyl moieties decreased it. It is important to add that PrP^res^ is a disease-promoting PrP^Sc^-like prion protein generated in vivo, following PrP^Sc^-induced misfolding of recombinant PrP^C^ [19].

Ouidja et al. [18] also observed that increasing polyanions size increases product efficacy. When considering the same degree of sulfate substitution, a maximum effect was found at 250 gly/chain in cell assays, with the partially sulfated HM_250_S_1.0_ (EC_50_ = 0.01 μΜ in ScGT1-7 cells). The product substituted with a phenylalanine methyl ester (X = PhMe) with formula HM_250_C_0.5_S_0.5_X_0.3_ increased the ability to inhibit PrP^res^ accumulation in ScGT1-7 cells compared with its non-hydrophobized analog (EC_50_ = 0.6 vs. >10 μΜ). The more voluminous and aromatic PhMe derivative was the one presenting the best effect, suggesting that it offers a hydrophobic binding point to the C-terminal fragment of the PrP^C^, rich in hydrophobic residues.

**Scheme 1:**
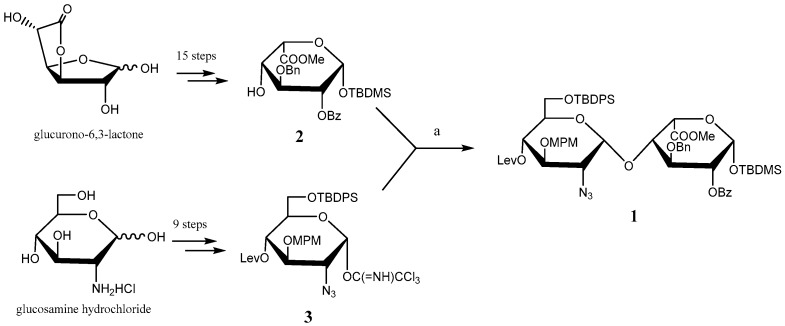
Synthesis of disaccharide **1**. *Reagents and conditions*: (a) TBDMSOTf, DCM, MS 4 Å, −20 °C → r.t., 18 h, 77%.

On the other hand heparin analogs may be structurally based on saccharide units whose disaccharide precursor GlcNS6S-IdoA2S **1** is presented in Scheme 1. In an attempt to know whether this disaccharide unit has sufficient anti-prion activity in prion-infected cells, recently, Teruya et al. [20] have characterized the binding profile of the representative structure of unprotected GlcNS6S-IdoA2S **1** to PrP. The synthesis of disaccharide **1** has been described by Saito and co-workers [21] using glucurono-6,3-lactone and glucosamine hydrochloride as starting materials to obtain the disaccharide building block by reaction of the sugar acceptor **2** with the conveniently protected 2-azido glycosyl donor **3** catalysed by *tert*-butyldimethylsilyl trifluoromethanosulfonate (TBDMSOTf). Acceptor **2** and donor **3** were synthesized over 15 steps from d-glucurono-6,3-lactone, and over 9 steps from glucosamine hydrochloride, respectively (Scheme 1).

Teruya et al. described a significant binding of unprotected disaccharide GlcNS6S-IdoA2S **1** with recombinant prion protein (PrP) (K_D_ = 9.7 μM), that was confirmed by competitive inhibition using heparin or pentosane polysulfate by surface plasmon resonance. However, the disaccharide structure did not exhibit anti-prion activity in prion-infected cells.

**Figure 2:**
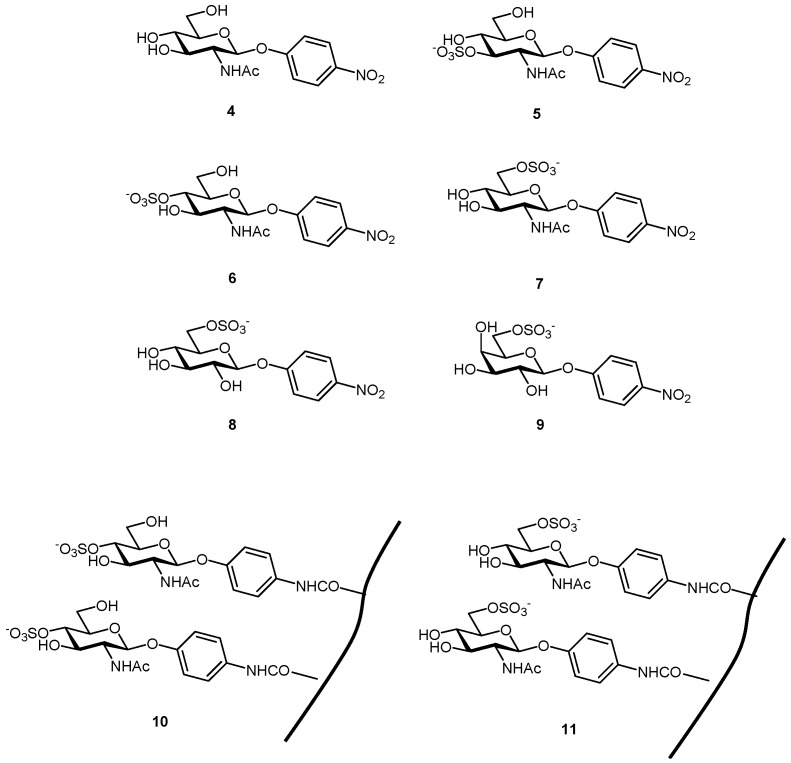
Compounds tested for the inhibition of PrP^Sc^ formation in prion-infected cells: sulfated *p*-nitrophenyl glycopyranosides, and glycopolymers composed by a polyacrylamide chain decorated with sulfated 2-acetamido-2-deoxy-β-d-glucopyranosyloxyphenyl moieties via amide bond (adapted from [26]).

Glycosaminoglycans (GAGs) are biomolecules of interest for prion-related diseases playing important roles in prion formation and infection [22]. GAG involvement in prion disease pathogenesis and PrP^C^ conversion into PrP^Sc^ has been widely reported [15,23,24,25]. Yagamuchi et al. [26] described, for the first time, polymers of acrylamide decorated, via an amide bond, with regioselectively sulfated 2-acetamido-2-deoxyglycopyranosyloxyphenyl moieties (Figure 2), and tested them, as well as the *p-*nitrophenyl sulfated glycoside precursors mimicking GAGs monomers, to screen for their ability to inhibit PrP^Sc^ formation in prion-infected cells.

Among the sulfated glycopyranosides and the polymeric compounds examined, the 4-sulfated derivative **6**, and two glycopolymers, **10** and **11** (Figure 2), inhibited PrP^Sc^ formation with 50% effective doses below 20 μg/mL (ED_50_ = 10, 4 and 9 μg/mL, respectively). A combination of an *N*-acetyl group at C-2 and a sulfate group at either C-4 or C-6 on glucopyranoside might be involved in the inhibition of PrP^Sc^ formation. Furthermore, PrP^Sc^ formation was inhibited by polymeric compound **11** but not by the glucoside **7**, suggesting the importance of a polyvalent structure.

**Scheme 2:**
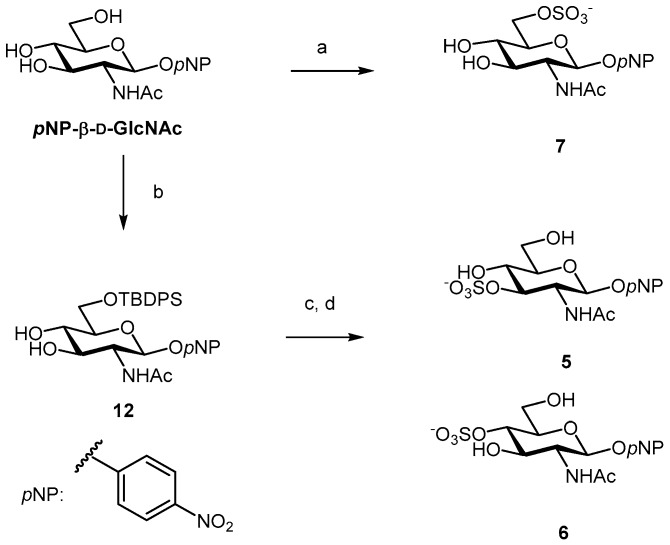
Synthesis of compounds **5**–**7**. *Reagents and Conditions*: (a) Me_3_N·SO_3_, DMF, 40 °C, 3 h, Dowex (Na^+^), 52%; (b) TBDPSCl, py, DMAP, r.t., 24 h, 95%; (c) Me_3_N·SO_3_, DMF, 40 °C, 10 h, Dowex (Na^+^), 63% (**5**/**6** = 1.4/1); (d) TBAF, THF, r.t., 24 h, 98%.

The synthesis of the *p*-nitrophenyl glycosides sulfated at 3, 4 or 6 position is depicted in Scheme 2 [27]. Position 6 of *p*NP-β-d-GlcNAc was selectively sulfated with a sulfur trioxide complex (Me_3_N·SO_3_) affording compound **7** in 52% yield. In order to generate sulfated glucosides at position 3 and 4, *tert*-butyldiphenylsilyl chloride (TBDPSCl) was chosen to firstly protect position 6. After sulfation with sulfur trioxide complex both **5** and **6** regioisomers were obtained in 63% overall yield and then separated by column chromatography.

**Figure 3:**
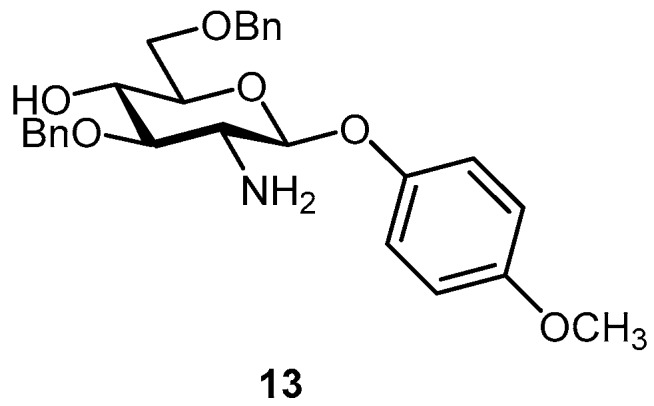
Molecular structure of the aminoglucoside **13**.

More recently Nishizawa et al. introduced the *p*-methoxyphenyl aminoglucoside **13** (Figure 3) as a new type of anti-prion compound, commercially available, and able to inhibit abnormal prion protein formation in prion-infected neuroblastoma cells in a prion strain-independent manner, when the cells were treated for more than 1 day. The 50% inhibition dose for PrP^res^ formation is 5.36 and 3.33 (μg/mL) in prion-infected ScN2a cells and N167 cells, respectively [28].

**Figure 4:**
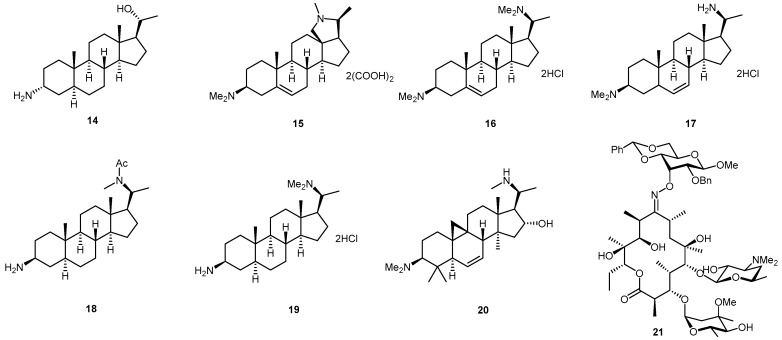
Chemical structure of inhibitors belonging to distinct chemical families: 3-aminosteroids **14**–**20** and erythromycin A derivative **21** [29].

In 2009, Charvériat and co-workers presented two different families of prion replication inhibitors [29]. One of them is based on 3-aminosteroid structures and the other on erythromycin A derivatives bearing saccharide residues and an oxime functionality (Figure 4). The eight compounds inhibited PrP^res^ accumulation in two cell cultures (SN56 and GT1 cell lines) infected by the Chandler scrapie strain, and by mouse-adapted scrapie strain 22 L, respectively.

Dose–response curves, with the identification of IC_50_ by densitometry, confirmed the concentration-dependent PrP^res^ inhibitory activity of compounds **14**–**21** on Chandler-infected SN56 cells [29]. Compound **17** and erythromycin derivative **21** showed the lowest IC_50_ (0.4 and 0.3 μM), respectively. Interestingly, they found that compound **21** interacted directly with PrP^res^ stability observing a change in the shape of the T_m_ curve in the fluorescence-based thermal shift assay. These results indicated that the interaction between PrP^C^ and compound **21** leads to a peculiar conformation of PrP^C^ that could induce alteration of its amyloidogenic properties, and consequently lead to the inhibition of conversion to PrP^res^ [29].

**Figure 5:**
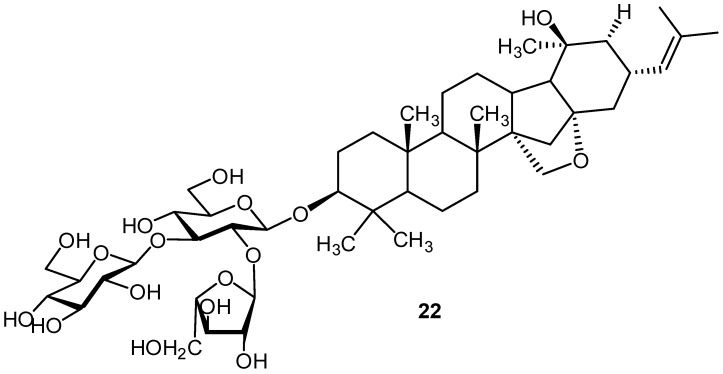
Molecular structure of bacoside A (**22**).

More recently other natural glycosides with anti-prion activity have been described in literature, namely bacoside-A (**22**, Figure 5). This natural triterpene glycoside bearing a trisaccharide moiety has been recently found to exhibit anti-amyloid properties [30]. It is an active principle of the medicinal plant *Bacopa monniera*, whose components include amphiphilic compounds structurally based on sterol glycosides, and is used in traditional Indian medicine to treat various nervous disorders, and to promote memory enhancement [31,32].

**Figure 6:**
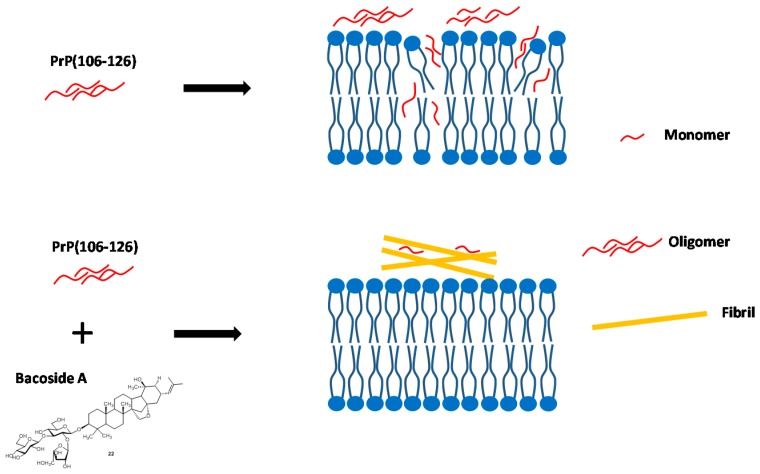
Schematic model for the activity of Bacoside-A; up: PrP_106–126_ alone forms abundant pre-fibril aggregates which interact with, and disrupt membrane bilayers; down: when PrP_106–126_ is pre-incubated with bacoside-A, fibrillation is accelerated resulting in lower abundance of membrane-active species (adapted from [30]).

Recent studies have suggested that bacoside-A might exhibit therapeutic effects against amyloid diseases, such as Alzheimer’s disease (AD) [33,34]. More recently Malishev et al. have investigated the interactions between bacoside-A and the 21-residue amyloidogenic determinant of the prion protein PrP_106–126_ by spectroscopy and microscopy experiments [35,36]. They evaluated the consequence of bacoside-A/PrP(106–126) interactions upon membrane bilayers (Figure 6). The experimental data revealed acceleration of PrP_106–126_ fibril formation in the presence of lipid bilayers. Importantly, they found that the enhanced fibrillation of the peptide, induced by bacoside-A, went in parallel with significant reduced membrane interactions and bilayer disruption, demonstrating a direct relationship between externally-induced accelerated fibrillation and inhibition of membrane interactions (Figure 6), indicating bacoside-A (**22**) as a potential therapeutic agent for TSE and amyloid diseases.

### 2.2. Aromatic Scaffolds with Nitrogen Containing Six-Membered Ring Heterocycles

#### 2.2.1. Pyridine Dicarbonitrile Derivatives

**Figure 7:**
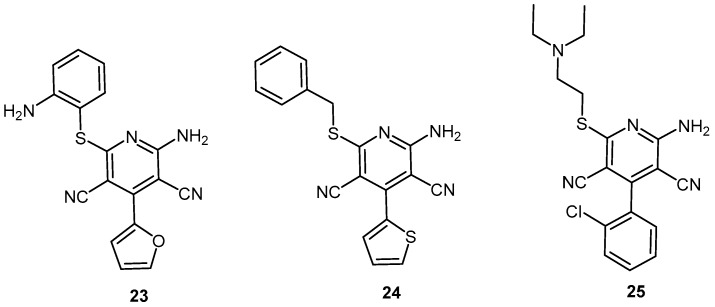
Three pyridine dicarbonitrile derivatives with in vitro anti-prion activity.

Pyridine dicarbonitrile derivatives were firstly published in 2000 as a new class of rationally designed leads with activity against PrP^Sc^ replication in PrP^Sc^-infected mouse neuroblastoma cells (ScN2a) [37]. In particular compound **23** (Figure 7) was reported to inhibit PrP^Sc^ formation in ScN2a cells in a dose-dependent manner, with an IC_50_ of 18.0 μM in ScN2a cells, and low levels of toxicity [37]. It has been proposed that it may bind to a chaperone required itself to bind to PrP for its conversion into PrP^Sc^, consequently inhibiting this conversion [37,38].

**Scheme 3:**
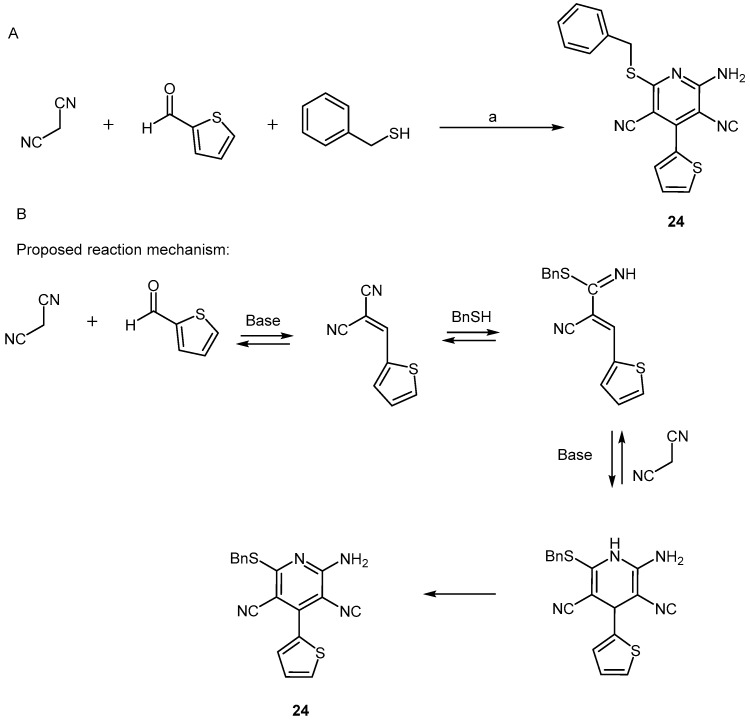
(**A**) One-pot synthesis of pyridine dicarbonitrile derivative **24**; (**B**) Proposed reaction mechanism. *Reagents and conditions*: (a) EtOH, reflux piperidine, 3 h, then air-exposed overnight (19%) [39].

In 2006, Reddy and co-workers focused on improving the activity of compound **23** through the development and evaluation of a small library of pyridine dicarbonitrile derivatives [39]. After performing docking studies with a proposed binding site model for compound selection, 45 structurally diverse compounds were synthesized by a one-pot three-component coupling reaction, herein illustrated in Scheme 3 for compound **24**, the most promising compound in this study.

Reaction optimization involved the study of the optimum aldehyde/thiol/malononitrile ratio, which was found to be 1:1:2, as well as the best solvent, the best catalytic base and the ideal reaction duration. Mechanistically, as depicted in Scheme 3B, during the first 3 h of reaction in ethanol under reflux, the initial base-catalyzed attack of a malononitrile anion to the aldehyde followed by elimination of water gives an adduct, which subsequently suffers the addition of benzylthiol to give an iminonitrile intermediate. Then, a second molecule of malononitrile is added to promote a cyclization reaction that affords the central dihydropyridine core; from this point, air-derived O_2_ is required for the aromatization into the final pyridine ring by chemical oxidation. Even though the yields obtained for this one-pot reaction were up to 52%, compound **24** was achieved in only 19% yield.

Compound **24** successfully interacted with all three PrP^C^ forms used in this study: human PrP^C^ (huPrP^C^; binding affinity at 40 μM, %RU_max_ = 55.5), truncated human PrP^C^ (t-huPrP^C^; %RU_max_ = 28.8) and murine PrP^C^ (moPrP^C^; %RU_max_ = 52.6). The analog possessing a 3-chlorophenylthiol at C-4 had a significantly higher affinity towards PrP^C^ (huPrP^C^ %RU_max_ = 123.6; t-huPrP^C^ %RU_max_ = 91.8; moPrP^C^ %RU_max_ = 72.2); however, it was found to be toxic. Nevertheless, compound **24** was more potent than the lead compound **23** in PrP^Sc^-infected mouse brain mesodermal (SMB) cells, and presented a high association/dissociation response ratio, which is ideal when looking for a potential new drug candidate.

Overall, most pyridine dicarbonitrile derivatives possessing the benzylsulfanyl moiety displayed some level of affinity towards PrP^C^. Through the evaluation of all synthesized analogs, Reddy and co-workers concluded that the substituent at C-4 of the central pyridine core of pyridine dicarbonitriles does not influence the binding to PrP^C^ as much as the substituent at C-6. In fact, this had been predicted in prior docking poses where the thioether moiety was placed deep inside the hypothetical pocket, whereas the substituent at C-4 was generally placed towards the opening of the cavity.

**Scheme 4:**
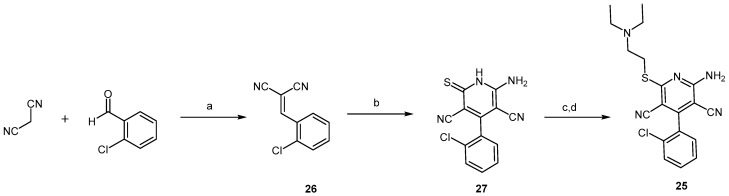
Synthesis of pyridine dicarbonitrile derivative **25**. *Reagents and conditions*: (a) β-alanine, EtOH, r.t., 72 h; (b) 2-cyanoacetamide, piperidine, EtOH, reflux, 8 h; (c) 10% KOH, DMF, r.t., 1 min; (d) (diethylamino) alkyl halide, r.t., 5 h [38].

Compound **25** (Figure 7) was published in a subsequent report by May et al. presenting a thorough structure-activity relationship study on pyridine dicarbonitrile derivatives bearing amine substituents at C-6 and furan or halobenzene substituents at C-4 [38]. The synthetic approach towards designed derivatives was quite distinct from the previous one, and is herein illustrated for compound **25** in Scheme 4.

Firstly, a β-alanine-catalysed Knoevenagel condensation afforded the arylidene malononitrile **26**, which was subsequently converted into intermediate **27** by reacting with a molecule of 2-cyanoacetamide, in the presence of piperidine, under reflux. Then, after treatment with 10% KOH in dimethylformamide (DMF), the haloamine was added to form the desired sulfanyl moiety, thus affording the final compound **25**. Although the yields for each reaction step are not specified, final compounds were obtained in 10–70% overall yield.

Most of these pyridine dicarbonitrile derivatives were not toxic up to 25 μM and exhibited EC_50_ values below 15 μM in ScNa2 cells. Moreover, they were generally more active than compound **24** (Figure 7) which, in this study, presented no activity up to 25 μM. Compounds with tertiary alkyl amines at C-6 were generally more active than those bearing primary amine moieties, whereas *meta*-substituted halobenzenes at C-4 were found to be preferable to furan-substituted compounds or to the corresponding *para*-substituted analogs. Among all synthesized pyridine dicarbonitrile derivatives, compound **25** was found to be the most promising, with an EC_50_ value of 5.5 μM in ScNa2 cells. Moreover, it presented high water solubility and a good permeability rate in a standard parallel artificial membrane permeability assay (PAMPA) (logPe = − 6.0 ± 0.1), indicating a good pharmacokinetic profile. However, no in vivo data are available up to this point.

#### 2.2.2. Piperazine Derivatives

**Figure 8:**
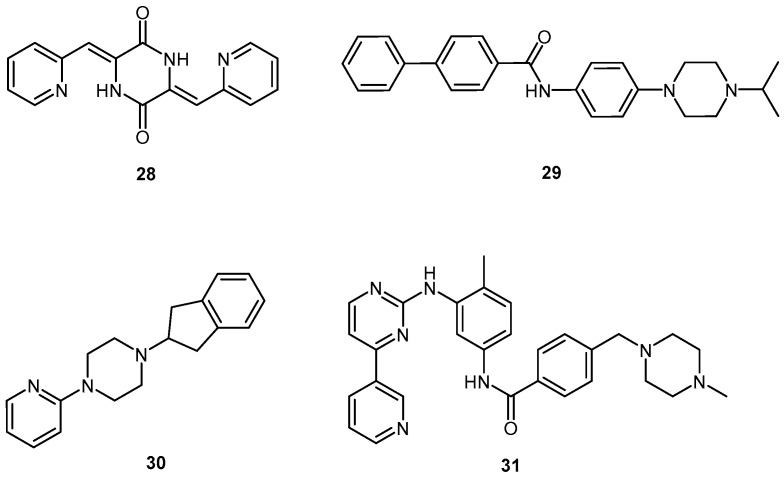
Four piperazine derivatives with anti-prion activity.

Based on the previously described neuroprotective activity and ability of the 2,5-diketopiperazine scaffold to modulate protein-protein interactions [40], Bolognesi and co-workers have generated a small library of compounds bearing different aromatic and heteroaromatic substituents in positions 3 and 6 of a central 3,6-dimethylpiperazine-2,5-dione core [41]. The authors envisioned that a planar, symmetrical molecule with aromatic end groups would be ideal to optimize the desired anti-aggregating properties and, in 2010, presented compound **28** (Figure 8) as a new anti-prion lead for further development.

Compound **28** inhibited PrP^Sc^ accumulation in PrP^Sc^-mouse hypothalamus (ScGT1) cells with an EC_50_ value of 4.1 μM, while maintaining approximately 75% cell viability at this concentration. Mechanistic studies focusing on aggregation kinetics revealed that this molecule acts by interacting directly with recombinant prion protein (recPrP) to inhibit its conversion into the pathological PrP^Sc^-like conformer and, interestingly, while the corresponding 3- and 4-pyridyl analogs were roughly ten times less active against PrP^Sc^ accumulation, compounds presenting benzene, furan, thiophene or quinolone moieties did not exhibit any activity.

**Scheme 5:**
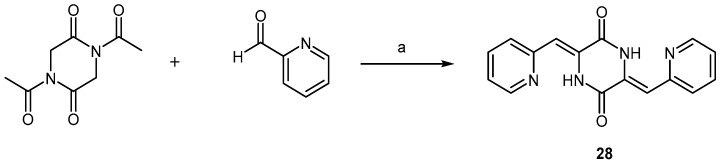
Synthesis of diketopiperazine **28.** *Reagents and conditions*: (a) TEA, DMF, r.t., 16 h (64%) [41].

The straightforward synthesis of compound **28** involved only one reaction step (Scheme 5). In this reaction, two equivalents of picolinaldehyde were mixed with one equivalent of the commercially available 1,4-diacetylpiperazine-2,5-dione dissolved in DMF, in the presence of triethylamine. A double aldol-condensation at room temperature gave compound **28** in 64% yield, affording exclusively the desired (*Z*,*Z*)-isomer.

**Scheme 6:**
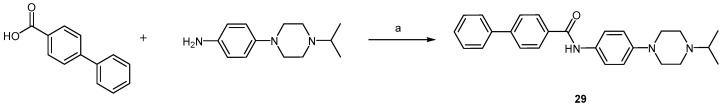
Synthesis of piperazine derivative **29**. *Reagents and conditions*: (a) HATU, DIPEA, anhydrous DMF, r.t., 12 h (66%) [42].

The isopropylpiperazine derivative **29** (Figure 8) was later published by Li et al. as the most promising compound of a small library of optimized arylamides with anti-prion activity, which were developed based on a moderately active piperazine arylamide derivative [42]. Here, the piperazine ring is placed in one of the extremities of the molecule, thus contrasting with Bolognesi’s rationale in which the diketopiperazine was the core moiety acting as the linker between two identical pyridine groups. The synthesis of this amide bearing the *N*-alkyl piperazine ring is very straightforward and involves only one reaction step, namely the standard coupling between a carboxylic acid and an arylamine carried out in the presence of the uranium salt *O*-(7-azabenzotriazol-1-yl)-*N*,*N*,*N*′,*N*′-tetramethyluronium hexafluorophosphate (HATU) as an acid activating reagent and *N*,*N*-diisopropylethylamine (DIPEA), in anhydrous DMF, at room temperature gave compound **29** in 66% yield (Scheme 6).

The initially conducted structure-activity relationship studies revealed that the biphenyl group linked to the carbonyl of the amide functionality led to more potent compounds than those bearing only one aromatic ring. The piperazine moiety was regarded as an important feature for the maintenance of compound solubility and thus was kept throughout this series of derivatives. Furthermore, 4-biphenyl analogs were preferable to their 3-biphenyl congeners, and compounds containing large *N*-alkyl moieties in the piperazine ring were more potent than those containing smaller *N*-alkyl groups. With an EC_50_ of only 22 nM against PrP^Sc^ accumulation in in PrP^Sc^-infected mouse neuroblastoma (ScN2a-cl3) cells, compound **29** stood out among this series of derivatives and was found to be 100-fold more potent than the original lead compound. It displayed acceptable metabolic stability in mouse liver microsomes (t_1/2_ > 60 min) and good brain exposure upon oral administration in mice, thus coming across as a promising lead for further development. However, the in vivo evaluation of this compound to confirm whether the nanomolar range potency observed in vitro is indeed transposable to TSE in vivo models is still lacking.

Compound **30** (Figure 8), on the other hand, was published by Leidel and colleagues as a piperazine derivative with activity in PrP^Sc^-infected mice [43]. This molecule was identified after high-throughput screening of 10,000 compounds in SMB cells, where four piperazine derivatives emerged as a new class of PrP^res^ conversion/amplification inhibitors. Noteworthy, a significant decrease in PrP^res^ formation was not achieved by compound **30** in a cell-free assay but it was shown to be very potent when tested in mouse neuroblastoma (N2a) cells, displaying an IC_50_ of 0.4 μM. Moreover, proteasome activity was not affected in its presence, indicating that it does not influence cellular trafficking or processing of PrP^C^. Taken together, these results suggest an indirect inhibitory mechanism of action for compound **30**, in which it probably targets misfolding or aggregation co-factors present in the cell-based assay, but absent when using only the purified prion protein. Alternatively, it may act by inducing cellular PrP^res^ clearance. Anyhow, this piperazine derivative successfully prolonged the incubation time in PrP^Sc^-infected C57BL/6 mice from 144 to 157 days when administered via an intraperitoneal injection, while a direct analog with a lower IC_50_ and bearing a 3-chlorophenyl moiety, instead of the 2-pyridyl group, did not.

This study succeeded to illustrate in vivo effects of piperazine derivatives against TSEs, and provided important hints on the potential anti-prion mechanism of action of these types of compounds. It is relevant to note that another piperazine derivative, compound **31** (Figure 8), was found to enhance the clearance of PrP^Sc^ in ScN2a cells, with an IC_50_ of 0.1 μM [44]. Interestingly, this compound is a well-known tyrosine kinase inhibitor indicated for the treatment of chronic myeloid leukaemia, and was shown to strongly activating the lysosomal degradation of PrP^Sc^, without affecting the biosynthesis or localization of PrP^C^. These results are consistent and clarify the findings regarding the mechanism of action of the piperazine derivative **30**.

**Scheme 7:**
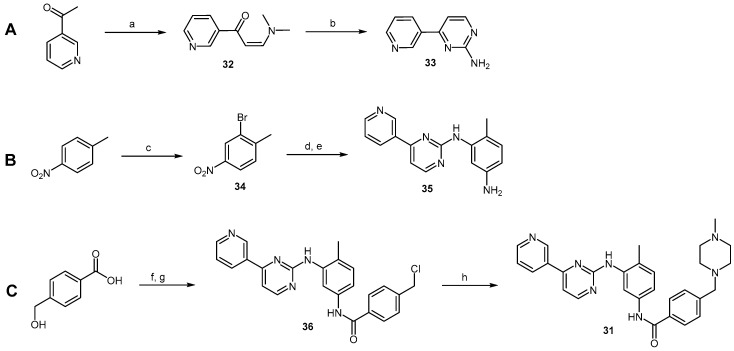
Synthesis of piperazine derivative **31** by the reaction steps presented in (**A**–**C**)**.** *Reagents and conditions*: (a) xylene, (CH_3_)_2_NCH(OCH_3_)_2_, reflux, 20 h (93%); (b) guanidine nitrate, NaOH, *n*-butanol, reflux, 12 h (86%); (c) Br_2_, Fe, 80 °C, 1.5 h (90%); (d) **33**, DMEDA, CuI, K_2_CO_3_, dioxane, 100 °C, 20 h (82%); (e) N_2_H_4_·H_2_O/FeCl_3_, MeOH, reflux, 6–8 h (84%); (f) SOCl_2_, DCM, reflux, 5 h (87%); (g) **35**, THF, TEA, 0 °C, 3 h (93%); (h) 1-methylpiperazine, reflux, 3 h (91%) [48].

Several possible synthetic routes for compound **31**, also known as 5TI571 or Imatinib, were reported since the early 1990s [45,46,47]. Scheme 7 shows one of the most recently published methodologies involving eight reaction steps with an excellent overall yield of 37% [48]. In this synthesis, the commercially available acetylpyridine was firstly enaminated and subsequently converted into the pyrimidyl amine **33** in the presence of guanidine nitrate (Scheme 7A). In parallel, after bromination of *p*-nitrotoluene, the so formed intermediate **34** was coupled to **33** using CuI as catalyst in a ligand assisted Ullmann-type *N*-arylation reaction. Subsequent reduction of the nitro group gave the arylamine **35** in 69% over two reaction steps (Scheme 7B). Finally, after full chlorination of 4-(hydroxymethyl)benzoic acid by SOCl_2_, compound **35** was coupled to the so formed acid chloride in the presence of triethylamine, affording amide **36** in 93% yield (Scheme 7C). Further *N*-alkylation of 1-methylpiperazine under reflux gave the final product, compound **31**, in 91% yield.

Even though piperazine derivatives did not show evidence of disease-modifying effects against TSEs so far, the above studies highlight some chemical features that may be important in future structure optimization approaches, namely the presence of an amide bond together with at least two aromatic rings and, conceivably, pyridine and pyrimidine moieties. However, additional in vivo studies are required for a better understanding of these requirements.

#### 2.2.3. Acridine Derivatives

**Figure 9:**
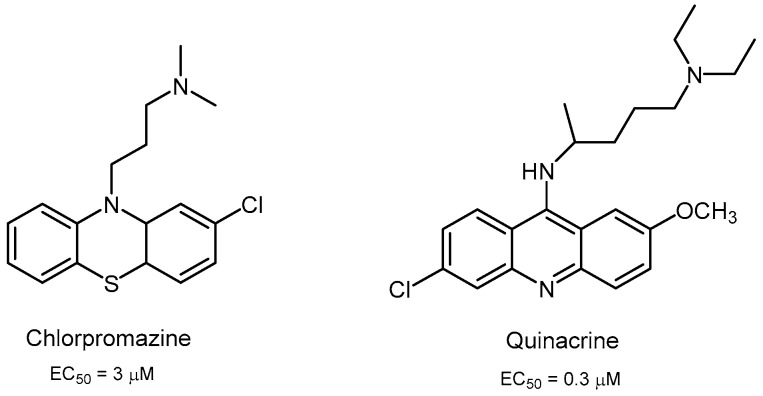
Structure of acridine analogs active for inhibition of PrP^Sc^ in scrapie-infected neuroblastoma (ScN2a) cells.

Quinacrine is one of the most known compounds presenting anti-prion properties and many analogs have been synthesized and studied over the last two decades. In 2001 Prusiner et al. reported that tricyclic derivatives of acridine and phenothiazine exhibited half-maximal inhibition of PrP^Sc^ formation at effective concentrations in scrapie-infected neuroblastoma (ScN2a) cells [49]. The EC_50_ values for chlorpromazine was 3 μM, whereas quinacrine was 10 times more potent (Figure 9), suggesting that they may be candidates for the treatment of CJD and prion diseases.

**Scheme 8:**
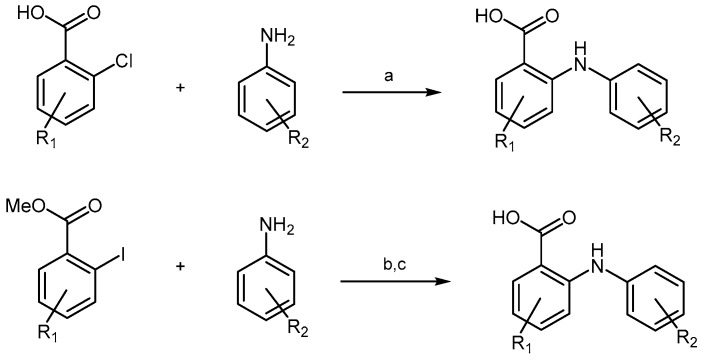
General synthetic routes for the synthesis of *N*-phenylanthranilic acids. *Reagents and conditions*: (a) Cu, py, amyl alcohol, (b) Pd(ac)_2_, DPEPhos, Cs_2_CO_3_, toluene, (c) aq. NaOH, acetone.

For the synthesis of quinacrine and their analogs, the corresponding anthranilic acids are used as starting materials by applying Ullmann-Jourdan reaction or Buchwald Hartwig amination [50] (Scheme 8). Copper-catalyzed Ullmann–Jourdan reaction [51] is applied to substituted 2-chlorobenzoic acids and the corresponding aniline derivatives yielding the anthranilic acids in moderate yields. However, the scope of the reaction is limited to electron-rich anilines and electron-deficient benzoic acids, leading to yield decrease. Using a palladium-catalyzed Buchwald–Hartwig amination seems to be a more robust method for the synthesis of anthranilic acids. Reaction of methyl 2-iodobenzoates and anilines using Pd(ac)_2_, bis-[2-diphenylphosphino)phenyl]ether (DPEPhos) and Cs_2_CO_3_ as base, and subsequent hydrolysis gave the corresponding acids [50] (Scheme 8).

**Scheme 9:**
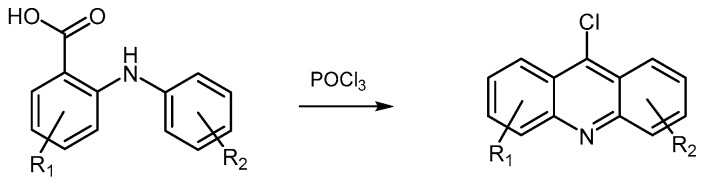
Ring closure procedure to achieve 9-chloroacridines.

In order to obtain the acridine scaffold, the resulting anthranilic acids are easily transformed into the corresponding 9-chloroacridines by reaction with POCl_3_ (Scheme 9).

**Scheme 10:**
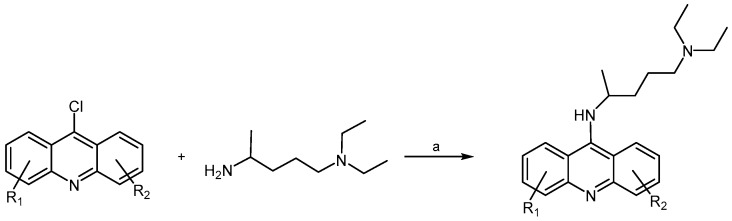
Synthesis of quinacrine derivatives. *Reagents and conditions*: (a) phenol, 100 °C.

Quinacrine derivatives are obtained from 9-chloroacridines and the corresponding amino spacer compound by reaction with phenol which acts as a catalyst at high temperature (Scheme 10).

In 2003, Vogtherr et al. identified the binding site of quinacrine at the C-terminal helix of human prion protein (hPrP) using NMR spectroscopy [52] Tyr225, Tyr226 and Gln227 residues of hPrP helix α3 turned to be the responsible of the interaction with quinacrine. K_D_ value was of 4.6 μM, 4 orders of magnitude weaker than the EC_50_ value found for PrP^Sc^ inhibition in the cell culture assay [49], probably due to a specific enrichment of this drug in cell organelles.

**Figure 10:**
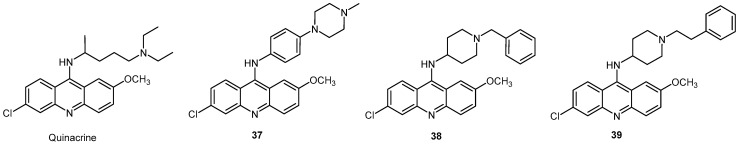
Quinacrine and analogs **37**–**39** with anti-prion acitivity against PrP^Sc^ in different cell lines.

In spite of the inhibition effectiveness of quinacrine, the lack of efficacy was attributed to inadequate accumulation of quinacrine in the brain, due to its removal by P-glycoprotein (Pgp) which is an efflux protein found in the blood brain barrier [53]. Indeed, when quinacrine was administered to prion-infected mice deficient in the genes encoding Pgp, brain levels of quinacrine were significantly increased [53,54]. Since quinacrine failed to extend the survival of prion-infected animals, authors speculated that continuous quinacrine treatment promoted the formation of drug resistant prions. This phenomenon could have contributed to the lack of in vivo efficacy of quinacrine. In spite of its limitations, quinacrine structural modification has yielded analogs with more potent in vitro anti-prion activity. Thus, other quinacrine derivatives with different substituents R_1_ and R_2_ and different spacers have been synthesized and their biological activity tested in the last two decades. Authors as Nguyen et al. showed that nature of side chain influenced compound cell-based potency, PAMPA permeability and binding affinity to hPrP_121–231_ when compared to quinacrine [55]. Several promising analogs were found with a more favorable anti-prion profile than quinacrine, in terms of potency and activity across different prion-infected murine cell models. Analogs **37**–**39** depicted in Figure 10 have shown sub-micromolar activities on several prion-infected mouse neuroblastoma cell lines, including the more resistant F3 where host cells were stably infected with a human prion strain. In particular, Nguyen Thi et al. found that piperazin analog **37** had EC_50_ values ranging from 0.1 to 0.7 μM on all cell models, being able to clear PrP^Sc^ at non-toxic concentrations of 1.2–2.5 μM, and presenting more activity than quinacrine in terms of EC_50_ values [56]. Behavior of analog **7** appeared to be unusual, showing EC_50_ values of 0.13 and 0.19 μM on ScN2a and F3 cell models respectively (Figure 10 and Table 1).

In general, potent and broad ranging anti-prion activities were found in the presence of side chains with aromatic residues substituted with basic functionalities. These structures also have shown a decreased effective permeability in PAMPA-BBB by increasing partition coefficient logD_7.4_, but kept within the permeability range of CNS compounds. Interestingly, compound **37** crossed the permeability P-glycoprotein assay (MDCK-MDR1) barrier with an efflux ratio that is half that of quinacrine (Table 1), indicating that it may be a weaker Pgp substrate.

Other quinacrine analogs with non-aromatic and basic side chains exhibited strong binding affinities for hPrP_123–231_ and good PAMPA permeabilities. Unfortunately, anti-prion activities were limited to ScN2a and did not extend to other cell models [55].

**Scheme 11:**
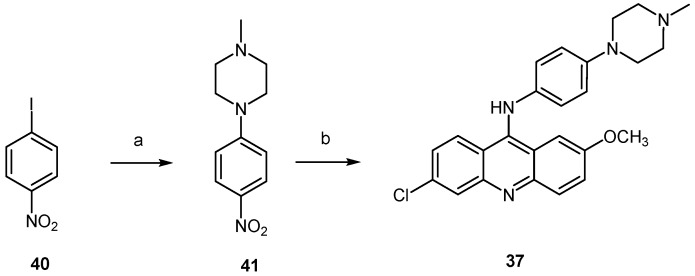
Synthesis of quinacrine analog **37**. *Reagents and conditions*: (a) amine, Pd(OAc)_2_, BINAP, Cs_2_CO_3_, anhydrous toluene, 120 °C, 95%; (b) (i) H_2_, Pd/C; (ii) 6,9-dichloro-2-methoxyacridine, ethanol, reflux, 24 h, 65% over the two steps.

The synthesis of analog **37** was carried out by a sequence of three steps (Scheme 11). Buchwald-Hartwig amination of 1-iodo-4-nitrobenzene with 1-methylpiperazine in the presence of a palladium acetate/2,2′-bis(diphenylphosphino)-1,1′binaphthalene (BINAP) catalyst generated compound **41** in 95% yield. Catalytic hydrogenation of the nitro group gave the corresponding amine to react with 6,9-dichloro-2-methoxyacridine in refluxing ethanol affording anti-prion compound **37** in 65% yield [56].

**Figure 11:**
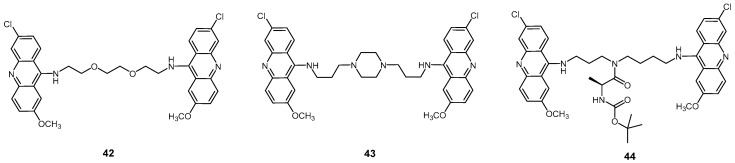
Bis(acridine) compounds **42**–**44** with anti-prion activity.

In 2003, Cohen et al. disclosed bis(acridine) analogs as an alternative to the acridine-based compounds [57]. They postulated that covalent dimers of quinacrine could be more potent inhibitors of prion replication due to an increased local concentration of the active moiety. Thus, dimeric acridine compounds were synthesized exhibiting a ten-fold higher activity than the respective monomeric compounds. The best results were obtained with the three bis(acridine) analogs **42**–**44** (Figure 11) that showed half-maximal inhibition of PrP^Sc^ formation in ScN2a cells at 40, 25, and 30 nM, respectively, and were not cytotoxic to uninfected neuroblastoma cells at concentrations of 500 nM.

**Scheme 12:**
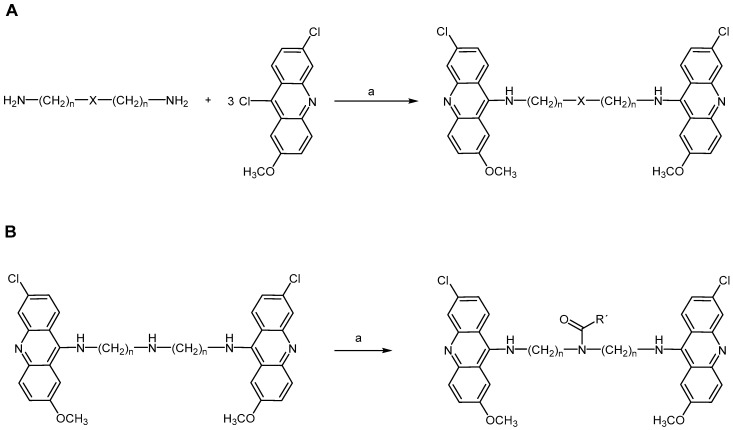
(**A**). General synthesis of bis(acridines). *Reagents and conditions*: (a) K_2_CO_3_, DMF; (**B**). Preparation of bis(acridines) bearing a central amide group. *Reagents and conditions*: (a) RCOOH, PyBrop, DIEA, DMF.

Structure–activity analysis revealed that spacer length and structure are determinant for inhibition of prion replication in cultured scrapied cells [57]. Compound **42** showed improved activity which could result from additional hydrogen-bonding interactions between the heteroatoms of the linker and the target receptor. Synthesis of bis(acridines) **42**–**44** [58,59] was carried out by reacting polyamines with 3 equiv. of 6,9-dichloro-2-methoxyacridine in DMF at reflux, in the presence of potassium carbonate as an inorganic base (Scheme 12A). In the case of compound **44**, a side chain central secondary amino group reacted with a carboxylic acid in the presence of bromotripyrrolidinophosphonium hexafluorophosphate (Py-BroP) to give the amide functionality [59] (Scheme 12B).

**Figure 12:**
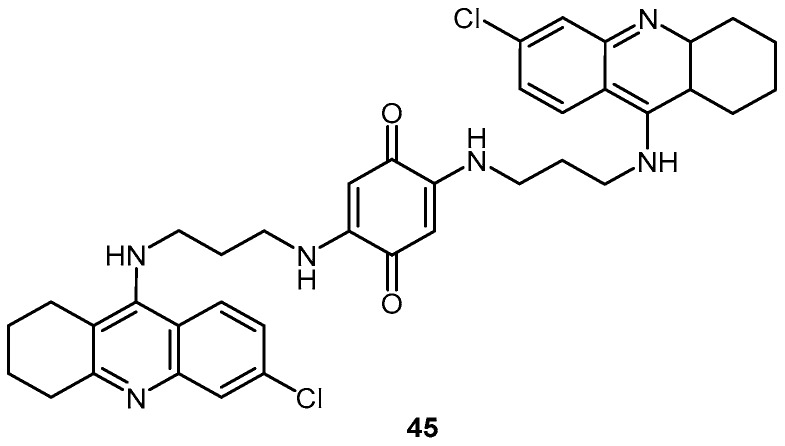
Compound **45** structure with EC_50_ = 0.17 μM on ScGT1 cells.

Bongarzone et al. [60] evaluated a small library of bis(acridine) analogs, bearing a 2,5-diamino-1,4-benzoquinone moiety as spacer component, against prion infection. The most active one acting on prion replication in the submicromolecular range was **45** containing the 6-chloro-1,2,3,4-tetrahydroacridine moiety (Figure 12), and showing an EC_50_ of 0.17 μM on ScGT1 cells, which was lower than that displayed by reference compound **42** (EC_50_ = 0.32 μM on ScGT1).

**Scheme 13:**
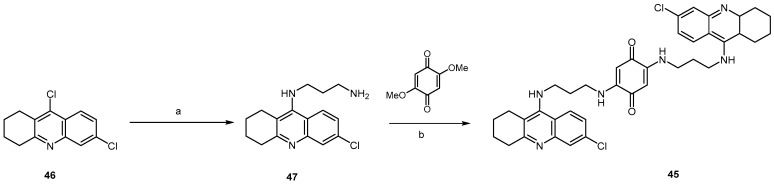
Synthesis of compound **45**. *Reagents and conditions*: (a) i. NaI, 120 °C (1 h); ii. Propane-1,3-diamine, 5 h, 120 °C, 50%; (b) EtOH, 5 h, 60 °C, 87% yield.

Compound **45** was synthesized starting from 6,9-dichloro-1,2,3,4-tetrahydroacridine by reaction with sodium iodide, followed by addition of a large excess of diamine at 120 °C for 5 h to generate the *N*-substituted diamine **47** in 50% yield (Scheme 13), whose reaction with 2,5-dimethoxy-1,4-benzoquinone at 60 °C for 5 h gave **45** in 87% yield [60].

**Figure 13:**
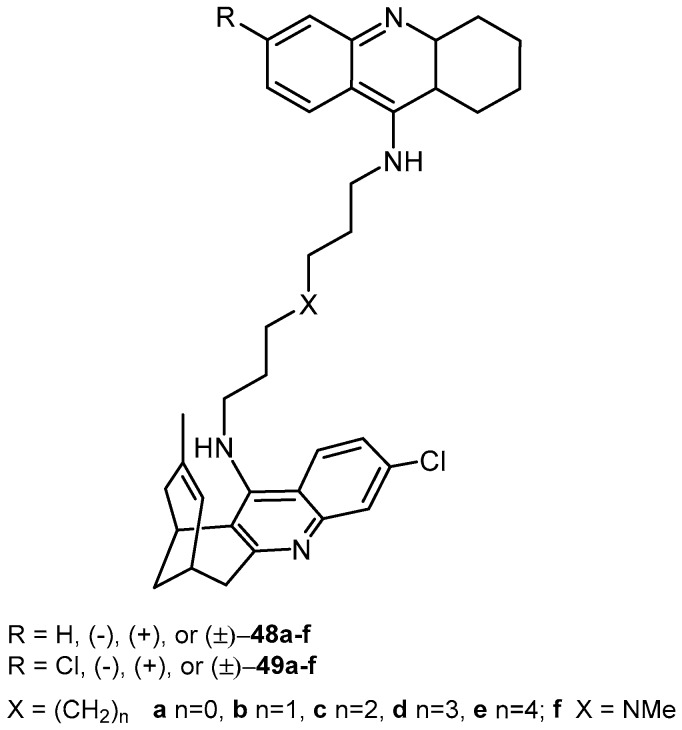
General huprine-tacrine heterodimer structure.

More recently Galdeano et al. [61] developed a family of huprine-tacrine heterodimers demonstrating a potent inhibition of human acetylcholinesterase (AChE) activity, in vitro neutralization of the pathological chaperoning effect of AChE toward β-amyloid peptide (Aβ) and prion protein aggregation. These heterodimers were able to cross the blood brain barrier in ex vivo experiments with OF1 mice. The general structure of these huprine-tacrine heterodimers is presented in Figure 13.

Enantiopure (−)-**48b** and (−)-**49b** showed a 5-6–fold more potent inhibitory effect (IC_50_ = 1.33 and 2.04 nM respectively) towards hAChE than their dextrorotatory homologs. All the huprine−tacrine heterodimers, at 100 μM, turned out to be potent inhibitors of the AChE-induced PrP_106−126_ aggregation with percentages of inhibition higher than 80% but no significant differences between compounds bearing an unsubstituted or chloro-substituted tacrine unit or between enantiomers were found.

**Scheme 14:**
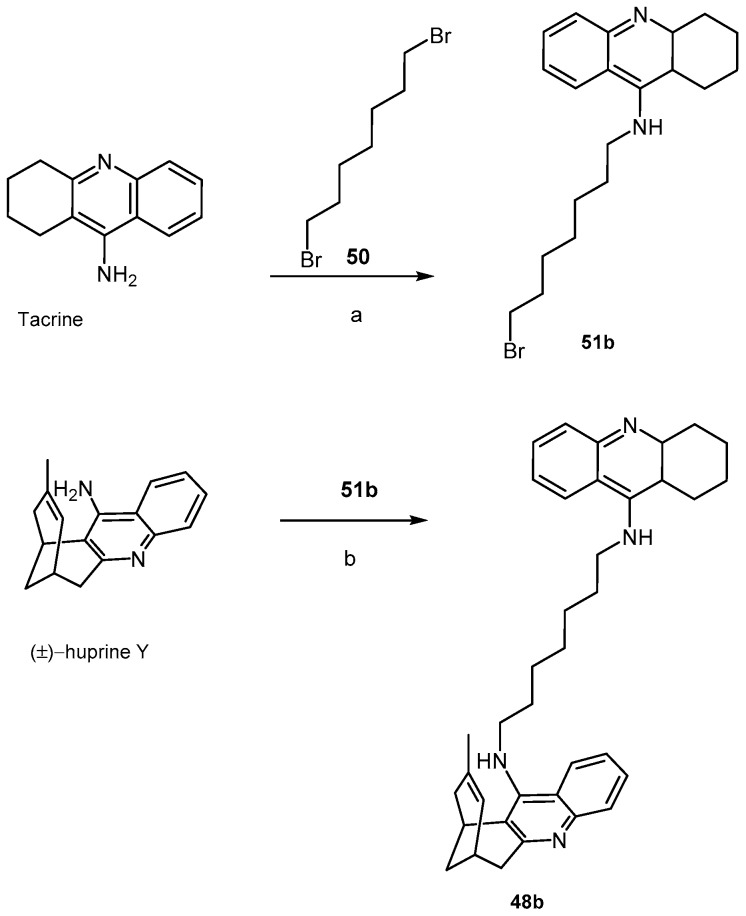
Synthesis of compound **48b**. *Reagents and conditions*: KOH, DMSO, 4 Å MS. (a) 2 h; (b) 3 days, 13% isolated yield over the two steps.

Thus, alkylation of tacrine [62] with 1,9-dibromoheptane **50** in the presence of KOH in DMSO [63] gave bromoalkyltacrine **51b**. Alkylation of racemic huprine Y with **51b** under similar reaction conditions afforded the heterodimer (−)-**48b** in 13% isolated yield [64] (Scheme 14).

Quinacrine has been reported to be an important prion inhibitor [65] However this compound did not increase the survival in a murine model of prion disease [49] and in high doses led to liver damage without therapeutic efficacy in humans affected with CJD.

**Figure 14:**
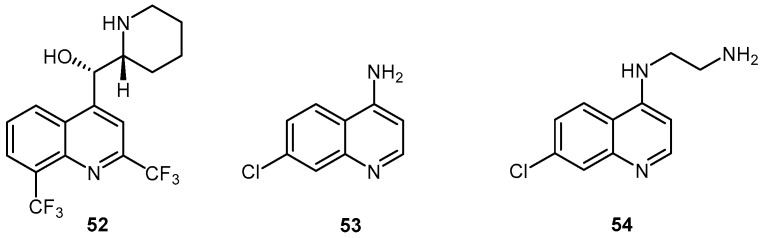
Aminoquinoline derivatives **52**–**54** with anti-prion activity.

Interestingly, a shared mode of action between acridines and aminoquinolines, whose core structure is embodied in acridines, has been discussed [66]. Aminoquinolines with anti-prion activity were also reported by Macedo and co-workers [67,68]. These aminoquinoline derivatives have been developed ten years earlier as potential antimalarial drugs. The hypothesis that the aminoquinoline scaffold could also act against PrP^Sc^ formation was based on a previous publication reporting the PrP^res^-inhibitory activity of the aminoquinoline derivative mefloquine (**52**, Figure 14), despite its lack of efficacy in Tg7 mice challenged with 263 K brain homogenates [69]. By studying these compounds, the authors aimed at gaining new insights on the minimum structure required for anti-prion efficacy, while trying to improve the therapeutic potential of both quinacrine and mefloquine. Compounds **53** and **54** (Figure 14) emerged as the most promising molecular entities with the aminoquinoline scaffold, with IC_50_ values of 0.2 μM and 0.1 μM, respectively, for the inhibition of the Syrian hamster prion protein ShaPrP_109–149_ aggregation.

By comparing the activity of all analogs studied by Macedo et al., it becomes clear that the primary amine present in both compounds **53** and **54** is an important feature for activity, particularly in the case of the latter [67]. In fact, while *N*-methylation of **53** led to an increase in ShaPrP_109–149_ aggregation from 8.4% to 38.6% at 1 μM, *N*-alkylation of compound **54** drastically increased the aggregation of ShaPrP_109–149_ to 82%. Moreover, the replacement of the primary amine by a hydroxy group also led to a dramatic decrease in activity, thus excluding the role of the primary amine as a hydrogen bond donor or acceptor in the affinity of these types of compounds towards the target protein.

These compounds definitively need to be further investigated to be considered as anti-prion leads for further development. Indeed, as evidenced by the studies presented and discussed in previous sections, the best inhibitors of PrP aggregation in cell-free assays are not necessarily the best compounds in cell-based assays, demonstrating that in vitro activity does not assure significant in vivo effects.

### 2.3. Aromatic Scaffolds Bearing Five-Membered Ring Heterocycles as Core Structure

#### 2.3.1. Diphenylpyrazole and Analogs

**Figure 15:**
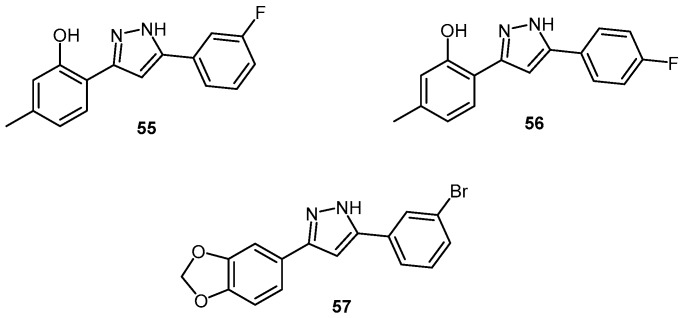
Three diphenylpyrazole derivatives (**55**–**57**).

In addition to piperazine derivatives (**30**, Figure 8), Leidel and co-workers identified diphenylpyrazoles as another class of promising compounds with anti-prion activity in vivo [70]. In a high throughput screening study against SMB and ScN2a cells conducted in 2011, compound **55** (Figure 15) stood out, exhibiting IC_50_ values of 0.6 μM and 1.2 μM, respectively. When compared to similar compounds also evaluated in this report, the methyl group is recognized as a key motif for the enhanced activity in SMB cells, but not in ScN2a; conversely, when the fluorine atom is replaced by a bromine, the activity drops in both cell lines. Compound **55** did not influence PrP^C^ expression or proteasome activity in ScN2a cells, and did not display any effect in a cell-free assay. The exact mechanism of action remains, for now, undisclosed. Yet, this diphenylpyrazole successfully prolonged the incubation time by 42 days in Tga20 mice challenged with the RML prion strain upon oral treatment, and increased survival time by 20 days in PrP^Sc^-infected C57BL/6 mice in a prophylactic approach. The *p*-fluoro analog **56** (Figure 15) was later tested on SMB cells and presented an IC_50_ of only 0.1 μM, but no in vivo studies have been published so far [43].

**Scheme 15:**
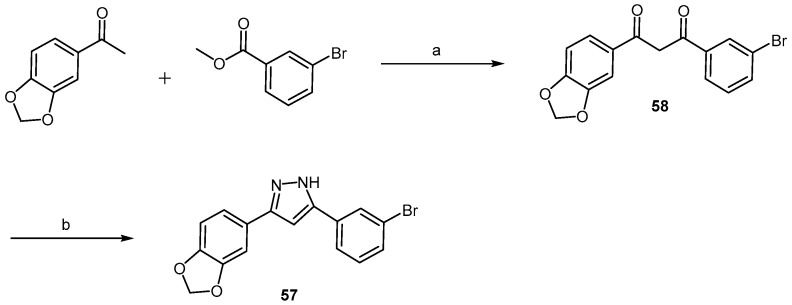
Synthesis of Anle138b (**57**). *Reagents and conditions*: (a) NaH, DMSO, THF, 10 °C, then r.t., 15 h (87%) (b) H_2_N-NH_2_·H_2_O, EtOH, reflux, 3 h (89%) [71].

Just two years later, compound **57** was identified by Wagner et al. another diphenylpyrazole with anti-prion activity [71]. After screening 20,000 structurally diverse compounds, the benzylidene-benzohydrazide unit came across as a potential lead chemical scaffold and, once optimized into the diphenylpyrazole core, a library of 150 diphenylpyrazole derivatives was prepared. The synthetic methodology is herein exemplified for compound **57**: briefly, after condensing the adequate ester and acetophenone starting materials in the presence of sodium hydride to afford de diketone intermediate, the pyrazole core is formed following treatment with hydrazine in ethanol, under reflux (Scheme 15). Compound **57**, also known as Anle138b, was obtained by this approach in 77% overall yield [71].

In this study published in 2013, Anle138b (**57**) was reported to be an amyloid oligomer modulator able to inhibit PrP^Sc^ formation in a cell-free protein misfolding cyclic amplification (PMCA) in vitro assay in 84%. Moreover, it was found to block the accumulation of PrP^Sc^ and neuronal cell death in mice infected with different PrP^Sc^ strains (EC_50_ = 7.3 μM for the murine RML prion strain or 7.1 μM for the vCJD human prion strain), while PrP^C^ expression was not affected.

Importantly, neuronal cell death was found to correlate with the rate of PrP^Sc^ amplification and not with the absolute levels of PrP^Sc^. Compound **57** permeates BBB, is orally available and, interestingly, it also inhibits the aggregation of α-synuclein (α-syn), an amyloid protein involved in the pathophysiology of Parkinson’s disease (PD). Indeed, amyloid proteins such as PrP^Sc^, α-syn and Alzheimer’s amyloid β 1–42 (Aβ_1–42_) share common structural features underpinning their similar aggregation-prone behavior [72]. This molecule was thus proposed to act transversally against amyloids, being potentially useful against different protein misfolding diseases [71].

Since then, Anle138b (**57**) has been and continues to be studied due to such promising results against amyloid protein aggregation. In agreement with Wagner’s results, it was found to increase survival in a mouse model of PD by 66 days [73]. Moreover, it showed affinity towards aggregated tau protein, which is implicated in the pathology of both PD and AD, while inhibiting tau aggregation in vitro and in vivo [74]. It prevented synapse and neuronal loss in tau transgenic PS19 mice, effectively increasing survival of these animals [74]. Hence, Anle138b seems to be not only a promising new lead against TSEs, but also against other diseases caused by amyloid proteins.

**Figure 16:**
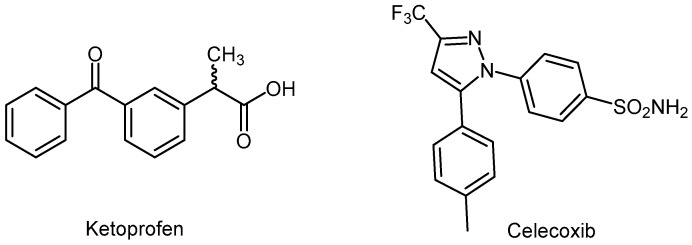
Structures of ketoprofen and celecoxib.

Villa et al. studied the neuroinflammatory role of ciclooxigenase (COX) activity, and its potential targeting for anti-prion therapies by comparing ketoprofen and celecoxib (Figure 16), preferential inhibitors of COX1 and COX2, respectively, on PrP_90–231_-induced microglial activation. Celecoxib, but not ketoprofen significantly reverted the growth arrest as well as NO and prostaglandine PGE2 secretion induced by PrP_90–231_, indicating that PrP_90–231_ pro-inflammatory response in microglia is mainly dependent on COX2 activation [75].

**Scheme 16:**
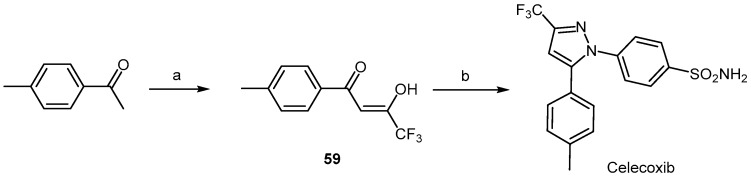
Synthesis of celecoxib. *Reagents and conditions*: (a) 25% NaOMe/MeOH, MTBE, ethyl trifluoroacetate, 94%, (b) (4-sulfamoylphenyl)hydrazine hydrochloride, EtOH, reflux 46%.

Synthesis of celecoxib is based on Claisen condensation of acetophenone with ethyl trifluoroacetate providing the adduct **59** in good yield. Reaction with (4-sulfamoylphenyl)hydrazine hydrochloride gave exclusively the desired regioisomer celecoxib in 46% yield [76] (Scheme 16).

#### 2.3.2. Thiazolamines and Oxazolamines

**Figure 17:**
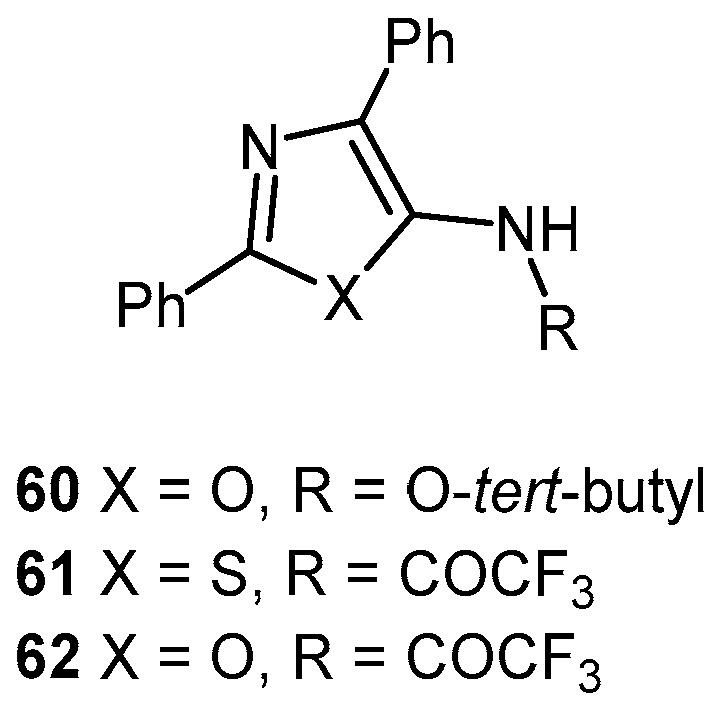
Oxazol-5-amine and thiazol-5-amine structures with anti-prion activity.

Thiazoles and oxazoles are found in the structure of a wide variety of biologically active molecules. In particular thiazol-5-amines are present in antibiotics [77] and photosensitisers [78]. Heal and co-workers [79] reported the first example of anti-prion activity in compounds of this type. Synthetic 2,4-diphenylthiazol-5-amine and 2,4-diphenyloxazol-5-amine derivatives **60**–**62** were found to bind hPrP^C^ and showed potent inhibition of PrP^Sc^ formation in infected SMB cells with EC_50_ in the range 1.5–20 μM (Figure 17).

**Scheme 17:**
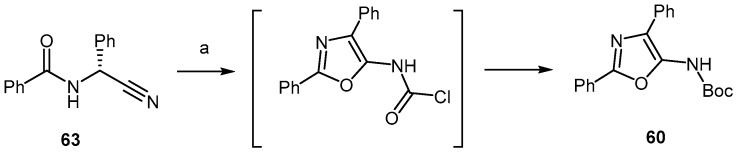
Synthesis of oxazol-5-amine derivative **60**. *Reagents and conditions*: (a) Triphosgene, DCM, r.t., 15 min, then *tert*-BuOH, DCM, r.t., 5 min, 22%.

Preparation of oxazole derivative **60** was carried out starting from 2-phenylglycinonitrile hydrochloride. Reaction with benzoyl chloride afforded the intermediate **63**, whose treatment with triphosgene and *tert*-BuOH permitted to obtain the target molecule in 22% overall yield (Scheme 17).

**Scheme 18:**
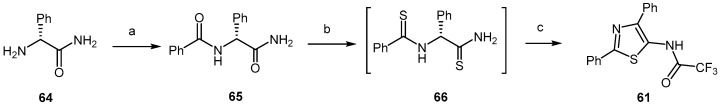
Synthesis of thiazol-5-amine derivative **61**. *Reagents and conditions*: (a) PhCOCl, py, NEM; (b) Lawesson’s reagent; (c) TFAA, DCM.

For the synthesis of thiazole **61**, d-(−)-phenylglycinamide **64** was reacted with benzoyl chloride in the presence of *N*-ethylmorpholine (NEM) to give *N*-acylglycinamide **65**. Treatment with Lawesson’s reagent to provide bis(thioamide) intermediate **45**, and then with trifluoroacetic anhydride (TFAA) gave **61** via a two-step one-pot procedure in 65% yield [80] (Scheme 18).

**Figure 18:**
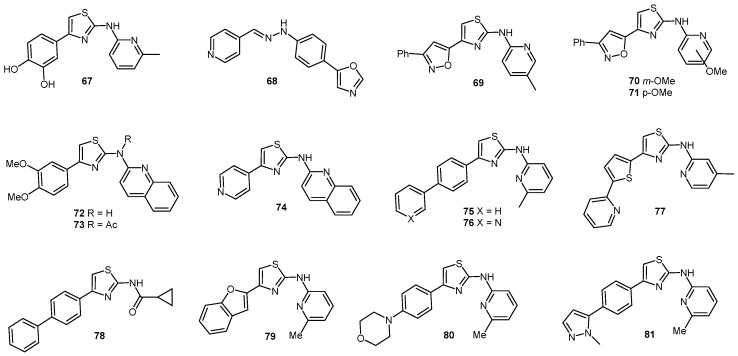
Structure of anti-prion thiazol-2-amines and oxazole **68**.

Thiazol-2-amines are another class of small molecules with anti-prion activity in prion-infected neuroblastoma cell lines. In Figure 18 the structure of some of the most potent anti-prion thiazol-2-amines is illustrated, and also oxazoles, namely compound **68**, used by some authors for comparison purposes because it was reported to produce a ≥50% extension of survival in treated animals. Prusiner and co-workers were pioneer identifying thiazol-2-amines as active compounds in ScN2a cells. The inhibition of PrP^Sc^ formation seemed to be the mode of action they postulated for these molecular entities [81]. *N*-(Methylpyridyl)thiazol-2-amines seemed to confer a more potent effect than analogs with unsubstituted pyridyl groups as suggested by SAR studies. In fact, compound **67** showed the more potent anti-prion activity with an EC_50_ of 2.5 μΜ in prion-infected neuroblastoma cell lines (ScN2a), than other tested analogs bearing *m*-dihydroxyphenyl and pyridyl groups.

Gallardo-Godoy et al. reported the identification of other thiazol-2-amine lead compounds that are orally absorbed and achieved high brain concentrations in animals [82]. In particular compound **69**, embodying also an isoxazole ring, displayed excellent stability to rat liver microsomes in vitro and exhibited an EC_50_ of 0.94 μΜ in prion-infected neuroblastoma cells (ScN2a-cl3), reaching a concentration of ∼25 μM in mice brain after three days of oral administration. The replacement of 4-methylpyridyl by 3-/4-methoxypyridyl resulted in more potent analogs, namely **70** (EC_50_ = 0.23 μM) and **71** (EC_50_ = 0.25 μM). Noteworthy, the analogs with a quinoline ring and a diprotected catechol moiety, namely **72** (EC_50_ = 0.11 μΜ, 10-fold more potent than its monocyclic version) and **73** (EC_50_ = 0.081 μΜ) were the most potent agents, even more than **74** bearing a pyridyl group. Also the presence of the hydrogen bonding donor, NH, was not determinant for anti-prion activity as shown by the activity of **73** when compared to that of compound **72** (Figure 18).

Ghaemmaghami et al. [83] reported that *N*-acyl substitution with small groups (e.g., acetamide, cyclopropylamide) was tolerated to keep the inhibitory effect, as confirmed with the activity of compound **78**. On the other hand aryl, heteroaryl or aliphatic rings appended to the aromatic ring attached to position 4 of the thiazolamine were tolerated, e.g., compounds **79** and **80**.

Regarding brain exposure evaluation, among the thiazoles embodying the quinoline moiety, compound **74** exhibited the highest brain AUC value (0.02 μΜ after 40 mg/Kg/day), while a higher brain exposure (1.31 and 0.88 μΜ after 40 mg/Kg/day, respectively) was found for **78** containing also the benzofurane ring and for **71** bearing the phenylisoxazole in its structure. However, thiazole **75** exhibited the highest brain AUC amongst the evaluated thiazolamines (8.70 μΜ after 40 mg/Kg/day). Even at the dose of 210 mg/kg/day, mice receiving **75** or **77** exhibited no adverse clinical or behavioral effects, suggesting that these compounds would be well tolerated on prolonged dosing in an animal model of prion disease. In general thiazolamine analogs exhibited higher exposure in brain than in plasma, suggesting that is not subjected to P-gp mediated efflux.

On the other hand, among in vivo efficacy studies published prior to 2013, arylhydrazone with the oxazole ring, compound **68**, was the only agent that produced a ≥50% extension of survival in treated animals compared to controls [84]. Based on this finding, Renslo et al. also determined the full PK profile of **75** and **77** compared to that of hydrazone **68** (Figure 18). They concluded that compound **75** achieved nearly 30-fold higher brain exposure following oral dosing than **68**, and also exhibited a much longer half-life, lower clearance, a superior brain/plasma ratio, and greater bioavailability. Compound **77** exhibited the highest volume of distribution of the three compounds. On the other hand **75** and **77** produced no signs of toxicity in mice. In contrast it was found that hydrazone **68** exhibited lethal toxicity at a dose of 150 mg/Kg/day.

Moreover, the replacement of the terminal phenyl ring in **75** with heteroaromatic or heteroaliphatic rings produced analogs with clearly superior potency in the ScN2a-cl3 assay. Thus, the presence of pyridyl in **76**, of morpholine in **80** and pyrazole in **81** led to analogs of **75** exhibiting EC_50_ values below 100 nM, a greater than 10-fold improvement in potency when compared to **75**. Compound **76** also exhibited excellent brain exposure in animals, yielding a greater than 20-fold improvement in brain AUC/EC_50_ ratio for **76** compared to **75**. More promising results were found with a series of cyclopropylamide substituted thiazole analogs, including compound **78**, the direct analog of **75**, that exhibited potency and in vivo brain exposure superior to **75**, with a brain AUC/EC_50_ ratio 10-fold higher than that of **75**.

However, thiazolamine-treated mice eventually showed accumulation of PrP^Sc^ in their brain and ultimately succumbed to disease. Compound **75** proved to be entirely ineffective against human CJD prions in susceptible transgenic mice expressing human PrP^C^. Prusiner and co-workers demonstrated that the eventual failure of thiazolamines can be accounted for by treatment-induced selection of thiazolamine-resistant prion strains [85].

To comprehend the mechanism of action of thiazolamines it has been suggested that they might interfere with the formation of new PrP^Sc^ in the cell, either directly by interference in the misfolding/assembly process or indirectly by modulating endogenous cellular clearance mechanisms [83]. However recent findings supported by X-Ray structure have shown that flexible, unstructured regions of PrP^C^ contribute to forming a cryptic small molecule binding site revealing direct interactions between thiazolamine and PrP^C^ [86].

**Scheme 19:**
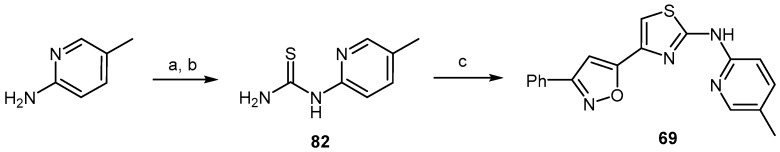
Synthesis of thiazol-2-amine **69**. *Reagents and conditions*: (a) PhSCN, acetone, reflux, 65%, (b) NaOH, MeOH, reflux; (c) 2-bromo-1-(3-phenylisoxazol-5-yl)ethanone, EtOH, reflux, 65%.

The synthesis of thiazol-2-amine analogs was carried out by the Hantzsch-type condensation of bromomethyl ketones with thioureas (Scheme 19). Synthesis of compound **69** started with reaction of the commercially available (5-methylpyridin-2-yl)amine with phenyl isothiocyanate in acetone under reflux, dissolved in MeOH and hydrolyzed by treatment with 1 N NaOH at 80 °C to afford thiourea **82** in 65% yield. Reaction with the commercially available 2-bromo-1-(3-phenylisoxazol-5-yl)ethanone under reflux for 4 h gave **69** isolated in 65% yield.

#### 2.3.3. Thiazolidines

Thiazolidines intervention in neuroinflammation-like responses, induced in microglia by PrP_90–231_, has also been explored. Microglia are CNS resident immune cells that comprise approximately 10–12% of glial cells in the brain and predominate in the grey matter. Although microglia form the first line of protection for neural parenchyma, and their uncontrolled activation may be toxic to neurons due to release of reactive oxygen (H_2_O_2_, or O_2_^−^) and nitrogen (NO) species, inflammatory cytokines, and prostaglandins (PGE2) [87,88,89]. AD patients show increased concentration of PGE2 in the cerebrospinal fluid and upregulated cyclooxygenase-2 (COX-2) in the brain; moreover, the deletion of PGE2 receptors result in neuroprotective effects in mouse models of AD [90,91]. Thus, being the activity of COXs the rate-limiting step in the conversion of arachidonic acid into prostaglandins, it was hypothesized that the neuronal injury induced by glial activation in AD and prion diseases can be reduced by non-steroidal anti-inflammatory drug (NSAIDs) treatment [92,93].

**Figure 19:**
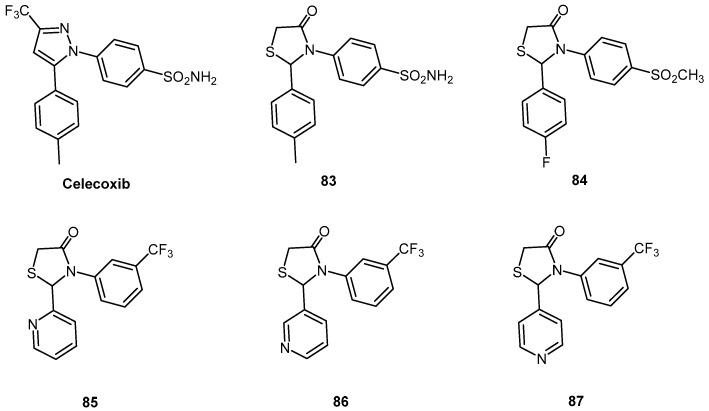
Chemical structures of celecoxib and the 2,3-diaryl-1,3-thiazolidin-4-one derivatives.

Recently, to study the possible role of NSAIDs in preventing amyloid-driven neuroinflammation, Villa et al. have demonstrated that PrP_90–231_ induces PGE2 release from microglia through the activation of COX-2, since this effect was selectively inhibited by celecoxib but not by ketoprofen, a prevalent COX-1 blocker [75]. Later on, the same authors searched for novel compounds able to inhibit glial cell activation induced by neuroinflammatory stimuli, such as PrP_90–231_ and lipopolysaccharide LPS. They tested a small library of 2,3-diaryl-1,3-thiazolidin-4-one derivatives structurally related to celecoxib (Figure 19), found to possess anti-inflammatory activity in animal models [94].

Thiazolidin-4-one **83** (Figure 19) inhibits microglia activation more efficiently than all other tested thiazolidines and celecoxib, whose structure embodies a pyrazole (see pyrazole section), lowering both iNOS and COX-2 activity, and reducing reactive oxygen species (ROS) release. These properties lead to the ability of **83** to revert neuroinflammation-like responses in mixed astrocyte and microglia induced by PrP_90–231_ and LPS.

**Scheme 20:**
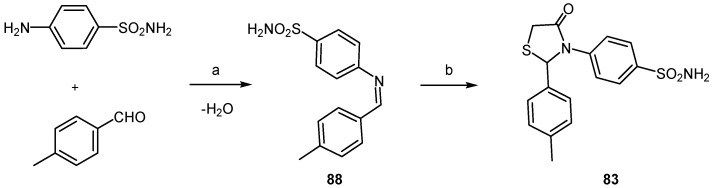
Synthesis of thiazolidin-4-one **83**. *Reagents and conditions*: (a) benzene, reflux, Dean-Stark apparatus; (b) HSCH_2_COOH, reflux, 36% (two steps).

Compound **83** was obtained by synthesizing the corresponding Schiff base **88** (Scheme 20) [95]. Reaction of 4-methylbenzaldehyde with 4-aminophenylsulfonamide under reflux followed by addition of α-sulfanylacetic acid maintaining the same conditions gave compound **83** in 36% overall yield (Scheme 20).

#### 2.3.4. Polythiophenes

**Figure 20:**
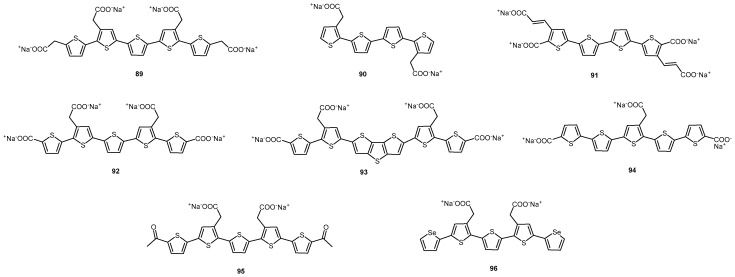
Polythiophene structures **89**–**96** studied in prion-infected mice.

Five-membered heterocycle ring polymers as thiophenes have been investigated as anti-prion compounds. Herrmann et al. [96] administered to the brain of prion-infected mice luminescent conjugated polythiophenes (LCPs) varying in the number of polyphene moieties, the number and distribution pattern of carboxyl groups (e.g., compounds **89**–**96**, Figure 20). They found that anti-prion activity required a minimum of five thiophene rings bearing regularly spaced carboxyl side groups. Solid-state NMR analyses and molecular dynamics simulations revealed that anionic side chains interact with complementary, regularly spaced cationic amyloid residues of model prions.

While five thiophene moieties were required for the minimal generic anti-amyloid LCP pharmacophore, as mentioned above, anionic side groups linked to the terminal thiophene rings is determinant for activity. Finally the periodicity of the anionic side groups also controlled the anti-prion properties, with the most effective pattern of regioregular charges being ca. 5 Å–10 Å–5 Å as in compound **89**.

**Scheme 21:**
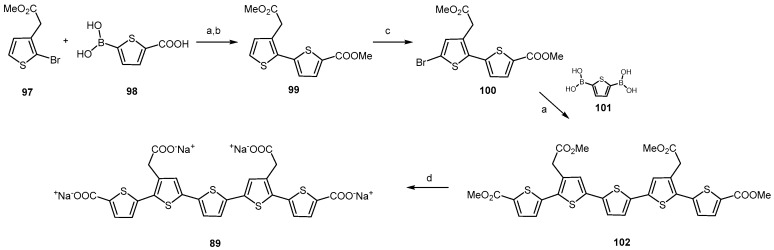
Synthesis of compound **89.** *Reagents and conditions*: (a) 1,4-dioxane/MeOH, PEPPSI^TM_^IPr, K_2_CO_3_, 70 °C, 20 min; (b) MeOH, H_2_SO_4_, 70 °C, 16 h; (c) NBS, DMF, 0 °C to r.t., 16 h; (d) NaOH (1M), 1,4-dioxane, 60 °C, 16 h.

Reduced PrP deposition was confirmed by immunohistochemistry in brainstems of mice treated with **89** and was associated to a conspicuous reduction in vacuolation and astrocytosis. In addition, the increased survival of **89**-treated mice, even when administered to preterminally scrapie-sick mice, suggested that LCPs may attenuate PrP^Sc^ toxicity, but confirmation remains open because it cannot be directly quantified. Compound **89** was synthesized [97] using the route represented in Scheme 21. Intermediate **99** was prepared by Suzuki cross-coupling conditions from brominated thiophene **97** and boronic acid **98**. After esterification, an overall yield of 71% over the two steps was obtained. Bromination of **99** with *N*-bromosuccinimide afforded the key precursor **100** in 73% yield, whose coupling to **101** yielded methyl ester pentamer **102**, giving compound **89** by hydrolysis, quantitatively (Scheme 21).

#### 2.3.5. Carbazoles

**Figure 21:**
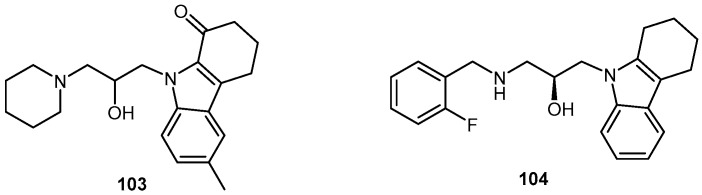
Two carbazole derivatives **103**, **104** with anti-prion activity.

Compounds **103** and **104** (Figure 21) are clearly distinct in terms of their chemical structure; yet, it is interesting to note the presence of key common elements in both structures: the number of hydrogen bond donors and acceptors, a nitrogen-containing five-membered ring, aromatic rings—one of which substituted with a fluorine atom, as well as a free hydroxy group. Compound **103** was also identified as a promising anti-prion scaffold by Kimura and co-workers in 2011 [98].

Compound **104** was developed inspired on compound **103** (Figure 21), previously reported for its affinity towards PrP^C^ while displaying an IC_50_ of 8.54 μM against PrP^res^ accumulation in GT1-7 cells infected with Fukoka 1 (FK-1) [98]. It was synthesized together with a library of fortyseven dihydrocarbazole analogs and derivatives, which were subsequently tested to allow a comprehensive structure-activity relationship study on the activity of the lead compound.

Among the most relevant conclusions drawn by the authors, the fused three-ring system was found to be crucial for the desired effect. Indeed, when the 1,2,3,4-tetrahydrocarbazole moiety was replaced by either a pyrrole, indole or 4,5,6,7-tetrahydroindole unit, the anti-prion activity was lost. Carbazoles and 2,3,4,9-tetrahydrocarbazoles, however, were able to retain the desired effects to the same extent as compound **103**. The hydroxy group was also found to be critical for activity by acting as hydrogen bond donor; what's more, by replacing the original piperidine by secondary amines such as the one in compound **104**, the anti-prion activity was further enhanced. The secondary amine function is indeed thought to interact with the target by acting as a hydrogen bond donor, while the hydrophobicity conferred by the *N*-linked substituent seems to be an important requirement. *O*-halobenzyl substituents in this position proved to be the most effective ones.

**Scheme 22:**
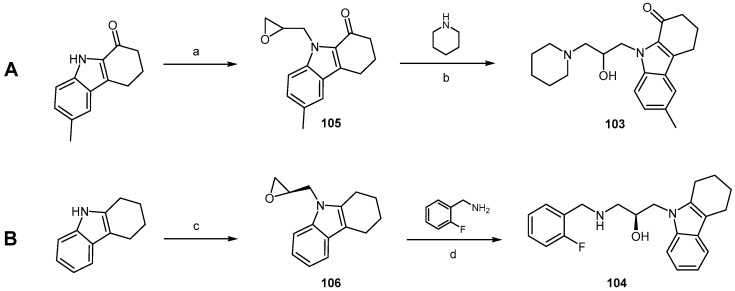
Synthesis of carbazole derivatives **103** (**A**) and **104** (**B**). *Reagents and conditions*: (a) (1) NaH, DMF, 0 °C, then 25 °C, 1 h; (2) epichlorhydrin, DMF, 0 °C, then 25 °C, 12 h; (b) EtOH, reflux, 12 h (51% over two reaction steps); (c) NaH, DMF, 0 °C, then 25 °C, 1 h; (2) *S*-epichlorhydrin, DMF, 0 °C, then 25 °C, 12 h; (d) EtOH, reflux, 12 h (60% over two reaction steps) [98].

Compounds were firstly prepared as racemic mixtures containing both (*R*)- and (*S*)*-*enantiomers, following a similar synthetic route here presented for the lead compound **103** (Scheme 22A). Herein, the starting material—in this case the 6-methyl-2,3,4,9-tetrahydro-1*H*-carbazol-1-one unit—was treated with sodium hydride followed by addition of epichlorhydrin in DMF at 25 °C. Subsequent epoxide opening by piperidine in ethanol then gave the final product in good overall yield [98].

**Figure 22:**
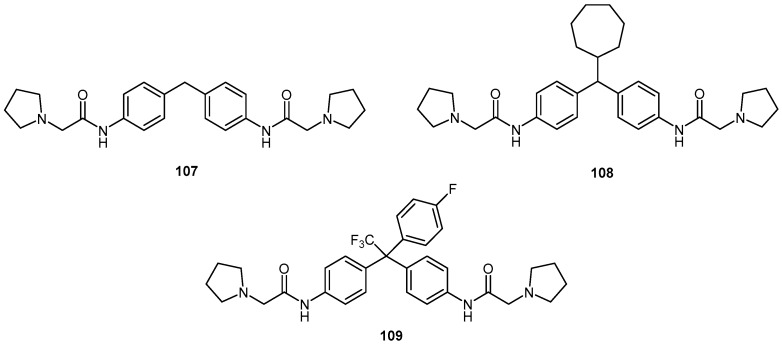
GN8 (**107**) and two derivatives **108**, **109** with anti-prion activity.

In order to compare the individual contributions of each enantiomer constituting the most active mixture tested in this study, (*S*)- or (*R*)-epichlorhydrin was, accordingly, used (Scheme 22B) [98]. Containing a 2,3,4,9-tetrahydro-1*H*-carbazole unit as the main core, and a *o*-fluorobenzyl group linked to the secondary amine, compound **104** then arose as the most promising one of this series, with an IC_50_ of 1.11 μM against PrP^res^ in GT1-7 cells infected with FK-1. It was found to be twice as potent as its (*R*)-enantiomer, eight times more potent than the lead compound **103**, and in vivo studies are currently taking place to assess whether it may, or not, deliver more encouraging results than those observed for some of the previously presented molecules. It would be interesting to compare those results, for instance, with the effects exhibited by GN8 (**107**, Figure 22) in vivo, since both compounds displayed identical IC_50_ values in the same cellular disease model.

#### 2.3.6. GN8 and Derivatives

*N,N'*-[methylenebis(4,1-phenylene)]bis[2-(pyrrolidin-1-yl)acetamide] (GN8, **107**, Figure 22) was discovered in 2007 by Kuwata and co-workers and, with an IC_50_ of 1.35 μM, significantly reduced the levels of PrP^Sc^ in a mouse neuronal cell line (GT1-7) infected with the Fukuoka-1 (FK-1) prion strain [99]. This compound also improved the incubation time of PrP^Sc^-infected mice up to 18 days when administered subcutaneously, and was found to stabilize PrP^C^ mostly by binding to its C-terminal domain and, possibly, by interacting with the C-terminal region of the GPI-anchored PrP^C^ [99].

**Scheme 23:**
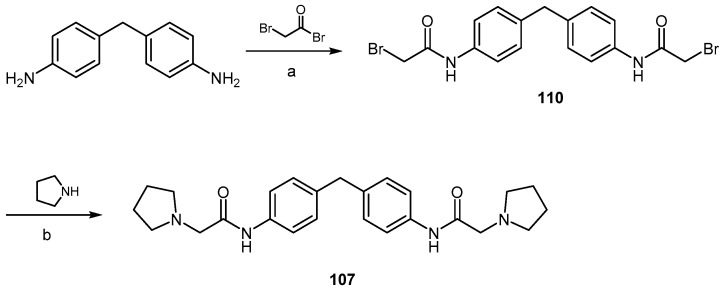
Synthesis of GN8. *Reagents and conditions*: (a) Py, DMAP, DCM, 0 °C, then 25 °C, 3 h (93%); (b) K_2_CO_3_, THF, 25 °C, then 60 °C, 12 h (88%) [100].

PrP^C^ is highly conserved in mammals and, therefore, GN8 is therefore strain-independent. This constitutes a relevant advantage in regard to previously described anti-prion compounds that act by targeting PrP^Sc^. Furthermore, this compound is easily accessed in only two reaction steps, with excellent overall yield (Scheme 23).

First, treatment of 4,4′-methylenedianiline with two equivalents of 2-bromoacetyl bromide in the presence of pyridine and dimethylaminopyridine (DMAP) gives intermediate **110**. Then, subsequent di-*N*-alkylation by pyrrolidine in THF at 60 °C affords the desired product [100].

**Scheme 24:**
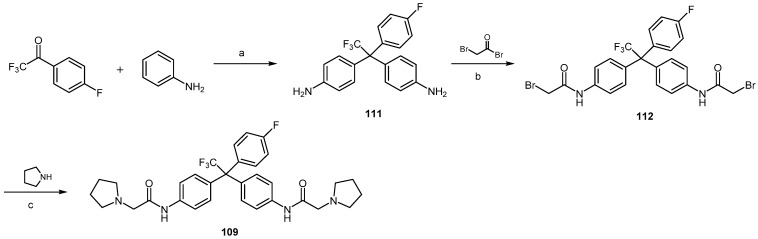
Synthesis of GN8 derivative **109**. *Reagents and conditions*: (a) PhNH_3_Cl, 25 °C, then 140 °C, 48 h (41%); (b) Py, DMAP, DCM, 0 °C, then 25 °C, 3 h; (c) K_2_CO_3_, THF, 25 °C, then 60 °C, 12 h (65% over two reaction steps) [100].

In 2011 Kimura et al. developed a series of GN8 analogs ultimately aiming at defining structural requirements for the activity of the lead compound [100]. Sixtyfour analogs were synthesized, two of which—compounds **108** and **109** (Figure 22)—displayed improved activity when compared to the prototype structure, both with an IC_50_ of 0.51 μM in GT1-7 cells infected with FK-1. The synthesis of these compounds involved an additional reaction step for the formation of the dianiline precursor, which is exemplified for compound **109** in Scheme 24. In this reaction, 2,2,2-trifluoro-1-(4-fluorophenyl)ethan-1-one was dissolved in a solution of aniline hydrochloride in aniline at high temperature to afford intermediate **111** in moderate yield. Then, as previously described in the synthesis of GN8 (Scheme 23), two consecutive *N*-alkylation reactions gave the final compound in 65% yield.

Overall, the conclusions of this study indicate that the structure of GN8 as a whole is essential for activity, as the deletion of either both pyrrolidines, or the full 2-(1-pyrrolidinyl)acetyl moieties conduct to a decrease in anti-prion activity. Moreover, *N*-methylation of the amide function leads to the same result. Indeed, it is supposed that the amide N-H groups and nitrogen atoms of the pyrrolidine units are crucial in the affinity towards PrP^C^, acting as hydrogen bond donor and acceptor groups, respectively. In contrast, the carbonyl groups are seemingly less important. All compounds in which the central methylene bridge in GN8 was replaced by other linkers, including compounds **108** and **109**, exhibited improved activity when compared to the lead structure. PrP^C^ levels were not affected by these compounds; however, conformational analysis showed that substituents at the benzylic position confer structural rigidity to the molecule, placing both phenyl groups in the right orientation and thus favouring the access to the binding site of PrP^C^. Yet, these substituents do not establish additional interaction points to the PrP^C^ structure. Compounds **108** and **109** did not present any toxicity at 2 μM in a cell-based assay and therefore constitute a promising class of anti-prion compounds for further development against TSEs.

### 2.4. Aryl Scaffolds Linked to Nitrogen Containing Functional Groups

#### 2.4.1. Congo Red Analogs

**Figure 23:**
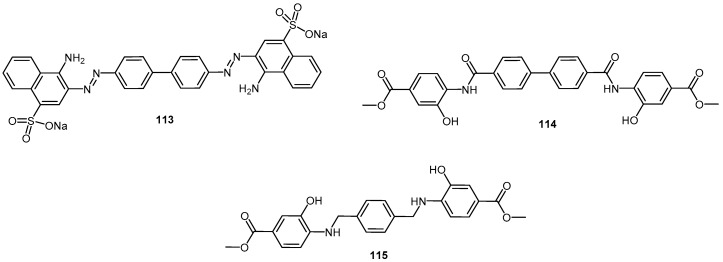
Congo red (**113**) and two analogs **114**, **115** with anti-prion activity.

Congo Red (CR, **113**, Figure 23) was found to inhibit the formation of PrP^Sc^ in cell-free assays back in 1983, and later to prevent the formation and accumulation of PrP^Sc^ in ScN2a cells [101,102]. However, it has a relevant lack of specificity and poor blood brain barrier permeability [103,104]. Importantly, the central benzidine moiety—which is released into the bloodstream after cleavage by gut and intestinal enzymes—has a relevant carcinogenic potential [105]. Since Rudyk and co-workers have shown that this moiety can actually be replaced by less toxic units without losing the desired activity, some research groups have joined efforts to design and develop new CR analogs with improved pharmacological profiles [106]. In 2004, Sellerajah and colleagues defined a strategy for the accomplishment of this task [107]. Previous work had already pointed towards both symmetrical parts of the lead molecule as being necessary for activity; additionally, the sulfonate groups could be replaced by carboxyl groups without causing a significant loss in activity [106,108]. Sellerajah and colleagues then envisioned that by replacing both diazo groups with bioisosteric equivalents such as amide bonds, the molecule could be prevented from being metabolized into the carcinogenic benzidine unit [107]. After synthesis and evaluation of a small library of CR analogs, compound **114** (Figure 23) was elected as the most promising for further development, being able to inhibit the formation of PrP^res^ in SMB cells with an EC_50_ value between 25 and 50 nM. It was significantly more efficient that the lead **113** and was effective in a PrP polymerization assay, which pointed towards a mode of action targeting PrP^C^ or the conversion between PrP^C^ into PrP^res^. Moreover, it displayed an IC_50_ value between 30 μM and 40 μM in a cytotoxicity assay against mouse cerebellar neuronal cells, which indicates that this compound has a good therapeutic window.

**Scheme 25:**
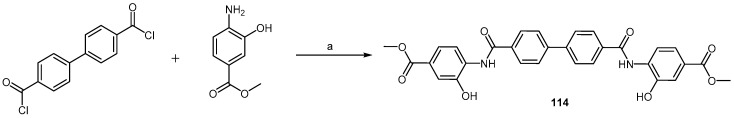
Synthesis of Congo Red analog **114**. *Reagents and conditions*: (a) TEA, anhydrous THF, 0 °C, 10 min, then rt, 18 h (14%) [107].

The synthesis of compound **114** was accomplished through a coupling reaction between (1,1′-biphenyl)-4,4′-dicarbonyl dichloride and methyl 4-amino-3-hydroxybenzoate in the presence of triethylamine, in anhydrous THF at room temperature (Scheme 25) [107]. Using this method, the target compound was obtained in 14% yield. The authors also described the synthesis starting from the dicarboxylic acid and using *O*-(benzotriazol-1-yl)-*N*,*N*,*N*′,*N*′-tetramethyluronium tetrafluoroborate (TBTU) and DIPEA in dimethylacetamide (DMA) at room temperature for 48 h. However, there was no significant improvement, as the yield was only 10%.

**Scheme 26:**
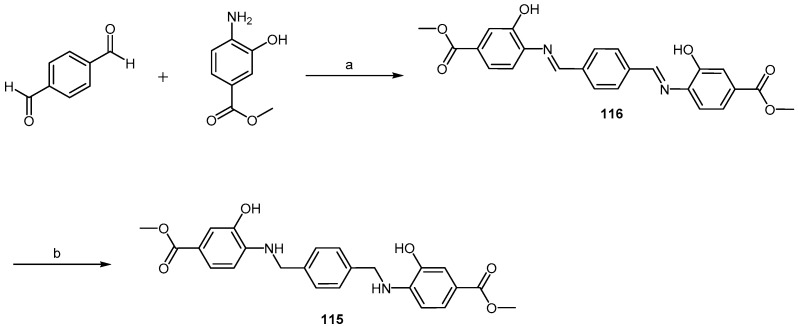
Synthesis of Congo Red analog **115**. *Reagents and conditions*: (a) EtOH, 55 °C, 5 h (76%); (b) DCM, NaBH_4_, MeOH, r.t., 12 h (49%) [107].

With an EC_50_ value of 75 nM, compound **115** (Figure 23) also exhibited tremendous potential in this study, and its synthesis, although involving one additional step, was much more effective than the previous two: after condensation of terephthalaldehyde with methyl 4-amino-3-hydroxybenzoate in ethanol at 55 °C to afford the imine intermediate **116**, a NaBH_4_-promoted reduction in DCM and MeOH at room temperature gave the desired product in 49% yield (Scheme 26) [107].

The ester moieties in both compounds **114** and **115** were initially conceived based on the hypothesis that they would be readily hydrolysed by esterase enzymes in the brain. Thus, the hydroxy groups were placed in the molecules to assure sufficient aqueous solubility [107]. However, the corresponding free carboxylic acid metabolites were not tested regarding their anti-prion activity in this study, and thus it is not known whether this rationale is, indeed, valid. Yet, in 2007, Webb et al. published a study showing the results of a set of mechanistic tests to verify the mode of action of both compounds, which were later named WSP677 and WSP740, respectively [109]. In this report, proteasome activity was shown to be increased by the interaction of these compounds with PrP. After 24 h, with one single treatment of 1 μM of compound SMB cells were “cured” based on the detectable levels of PrP^Sc^. Remarkably, CD-1 mice inoculated with SMB cell extracts containing compound **115** presented no symptoms after 400 days after inoculation. This implies that the agent of disease transmission was eliminated by the action of this compound. However, compound **114** did not exhibit the same effects. These data not only place compound **115** in a lead position for the development of new molecules for the treatment of TSEs, but also reflect the discrepancy between in vitro and in vivo effects when dealing with anti-prion therapies.

#### 2.4.2. Sulfonated Arylamides

**Figure 24:**
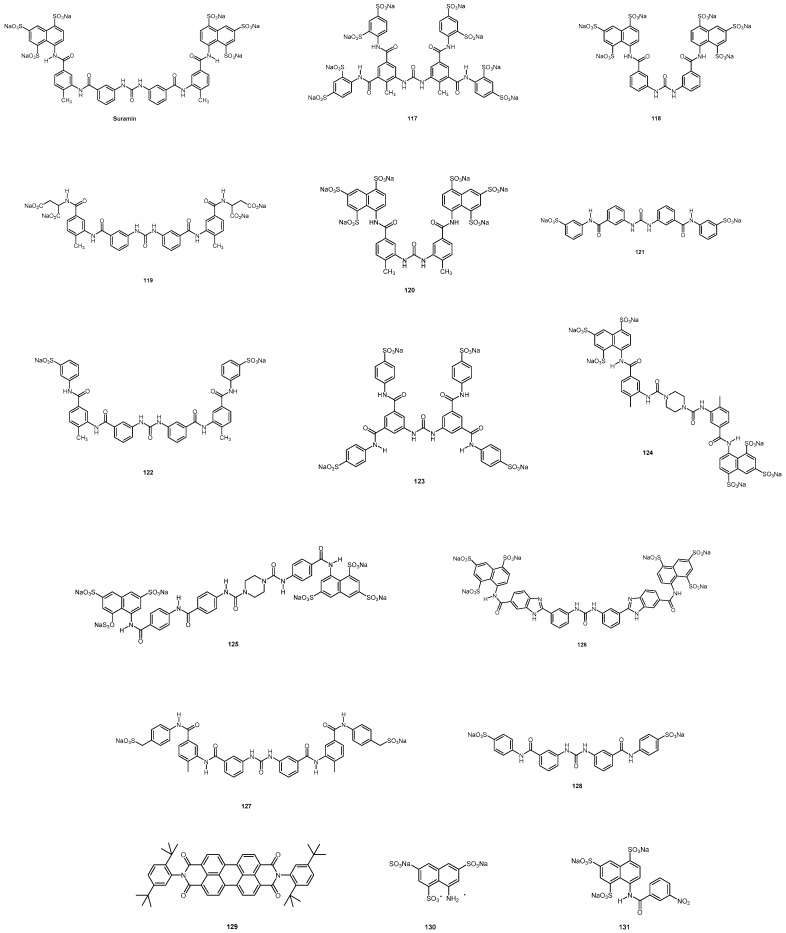
Suramin-related structures tested for anti-prion activity.

Suramin is a polysulfonated aromatic urea (Figure 24), discovered in the 60´s to treat African sleeping sickness [110]. More recently it has been described as modulator of PrP^C^ expression, with the ability to modify biochemical properties of PrP^C^ including solubility and its half-life [111]. Kiachopoulos and co-workers described how suramin induces misfolding of the cellular prion protein and interferes with the propagation of infectious scrapie prions [112]. Gilch et al. characterised the effects of suramin on prion biogenesis in cell culture and in vivo models. They reported suramin inhibiting de novo formation of PrP^Sc^ by inducing intracellular PrP^C^ aggregates which are targeted from Golgi/Trans Golgi Network (TGN) compartments to a lysosomal degradation pathway [113].

All these previous findings have led to the evaluation of new suramin-related compounds developed by Nunziante and co-workers [111] (Figure 24). Among the compounds tested, only those with symmetrical bipolar structure and sulfonic acid groups showed potent inhibition of PrP^Sc^ synthesis. Two half-molecules lacking symmetrical structure had no effect on PrP^Sc^ accumulation. Uncharged compounds did not have activity, despite their symmetrical aromatic structure. Effective compounds induced aggregation of PrP^C^ and reduced its half-life without affecting PrP^C^ cell surface expression, which was crucial for their activity. The effect of these suramin derivatives on PrP^Sc^ was determined in prion-infected neuroblastoma cells (3F4-ScN2a) using a solubility assay and proteinase K (PK) treatment followed by immunoblot analysis. The accumulation of PKresistant material was completely eliminated after treatment with suramin and with compounds **117**, **118**, **120**, **122**, **123**, **124**, **125**, and **126**. Substances with carbonic or phosphonic acid substitutions, **119** and **121** respectively, showed no activity or only mild inhibitory effects on PrP^Sc^. No PrP signal was detected in cells incubated with **128** owing to the strong toxicity of the compound. Asymmetric and uncharged drugs (**129**, **130** and **131**) were ineffective in reducing the amount of PrP^Sc^, and did not show any effect on PrP^C^ solubility.

On the other hand, when cells were subjected to a solubility assay, the immunoblot analysis revealed aggregation of PrP when cells were exposed to suramin and to compounds **117**, **119**, **122**, **123** and **124**.

Nunziante et al. [111] also examined the influence of suramin analogs on the cellular localization of PrP by confocal microscopy. After treatment of wtN2a cells with **117** or **118**, the PrP signal at the cell surface was still consistent with that detected in untreated cells. In addition, **117** and **118** also induced increased partitioning of PrP in intracellular compartments. This intracellular PrP population was mainly present in lysosomes. These results showed that, in contrast to suramin, these derivatives did not interfere with the cell surface expression of PrP^C^.

**Scheme 27:**
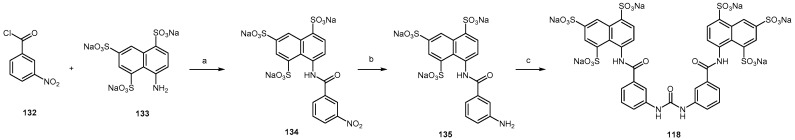
Synthesis of compound **118**. *Reagents and conditions*: (a) toluene, r.t,. 6 h, 83%, (b) Pd (10%) on charcoal, H_2_O, 4 bar H_2_, rt, overnight, 98%, (c) H_2_O, phosgene (20%) in toluene, r.t., 6 h, 85%.

As an example of arylamide synthesis, Scheme 27 shows the synthetic route to obtain compound **118** [114]. 3-Nitrobenzoyl chloride **132** was reacted with compound **133** in a biphasic mixture of toluene and water at acid pH for 6 hours affording the amide **134** in 83% yield. Reduction of nitro group by hydrogenation generated compound **135** in 98% yield. Finally, reaction of **135** with phosgene gave suramin derivative **118** in 85% yield.

### 2.5. Polyphenols

This structurally diverse family of compounds, the polyphenols, are often found as the major plant components responsible for their medicinal properties. Their bioactivity against neurodegeneration is mainly due to their antioxidant [115,116,117,118,119], anti-inflammatory [120,121], and anti-amyloidogenic [122,123,124] effects. Polyphenols mode of action includes inhibition of intracellular kinases activity [125], binding to cell surface receptors [126], and modifying cell membrane functions [127]. These natural drugs are potent therapeutic agents exhibiting pharmacological properties against Alzheimer’s disease and dementia, multiple sclerosis, ischemic stroke, Parkinson’s and Huntington’s disease [128].

**Figure 25:**
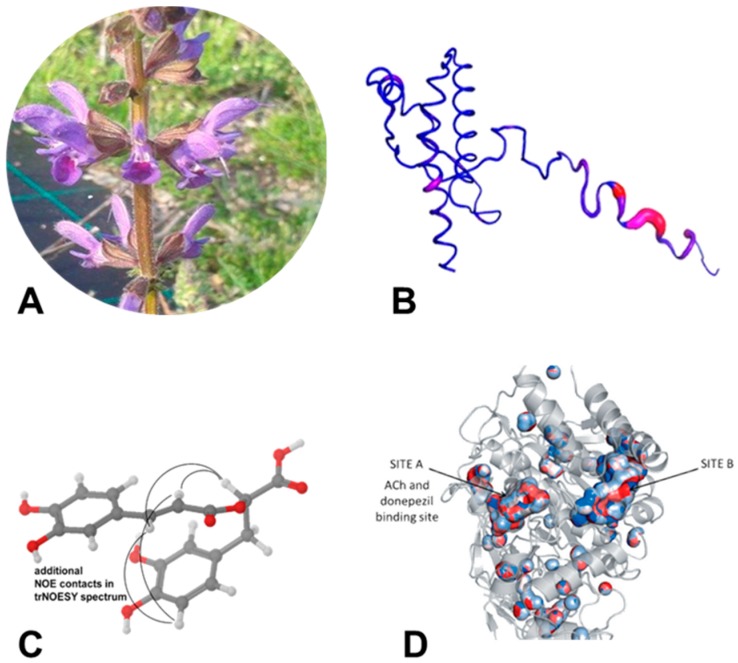
(**A**) *Salvia sclareoides*; (**B**) Structure of huPrP_91–231_ indicating residues affected by *S. sclareoides* butanol extract, where the color and thickness of the ribbon indicates increased interaction. Residues affected: 93–112 in the unstructured N-terminal region of PrP^C^, 130–187 comprising the two strands of the beta sheet, helix 1 and the beginning of helix 2, and 205–225 in helix 3 at the C-terminus of the structured domain [131]; (**C**) Rosmarinic acid interacts with Aβ_1-42_, and the aromatic protons are mostly involved in the binding, as detected by NMR experiments [132]; (**D**) Depiction of acetylcholinesterase binding site A for donepezil, a drug in the market to treat AD and that of the newly discovered binding site B for rosmarinic acid [133].

Rauter and co-workers reported the potent acetylcholinesterase (AChE) inhibitory activity of *Salvia sclareoides* extracts, a spontaneous plant growing in Iberia (Figure 25A) [129]. Plant extracts also delayed prion propagation in a cell-based screening assay [130], and the prion binding properties were then evaluated by NMR measurements [131]. The *n*-butanol extract turned out to bind to the cellular form of human prion protein in a specific manner, causing conformational perturbation in three different regions of the protein including the highly dynamic N-terminal region (Figure 25B). The authors found out that the major extract component was rosmarinic acid, and NMR measurements have shown its interaction with the Alzheimer’s disease (AD) amyloid oligomers Aβ1-42, with aromatic protons being mostly involved in the binding (Figure 25C) [132]. Interestingly, a new binding site of rosmarinic acid to AChE was also discovered by the authors, namely the binding site B in Figure 25D [133], opening the way to the development of new drugs based on rosmarinic acid as scaffold, aiming to innovate the standard therapy to treat AD patients with donepezil, rivastigmine and galantamine. The search for new AChE inhibitors is encouraged as this cholinesterase is able to accelerate amyloid formation [134]. Indeed PrP^C^ decrease also accelerates Aβ formation and it was reported that its fate in ageing may be related to the mechanisms involved in neurodegeneration, affecting also AD [135], but the role of PrP^C^ on AD is not yet fully understood. Noteworthy, prions are amyloid forming proteins and the discovery of anti-amyloidogenic compounds against AD may also be an added-value discovery for the combat of prion diseases.

Caughey et al. screened 2000 compounds covering drugs and natural products for the inhibition of PrP^Sc^ in ScN2a cells infected with scrapie strain RML or 22L [136]. While 40 compounds showed IC_50s_ below 10 μM, 17 of them, depicted in Figure 15, were the most potent ones, having IC_50s_ below 1 μM against both strains. It was also found that many of them were natural without observed toxicity. Quinacrine and lovastatin were previously identified as PrP^Sc^ inhibitors [137,138]. Tannic acid **84**, a constituent of foods, turned to be the most potent inhibitor, even better than quinacrine, with IC_50_ ~100 nM.

The compounds were also evaluated in a solid-phase cell-free (SP-CFC) hamster PrP conversion assay. Only polyphenols **136**, katacine **137** and 2′′′-bisepigallocatechin digallate **138** inhibited the cell-free reaction, and their IC_50_ values were near 100 nM. Epigallocatechin **139** and epicatechin **140** were not PrP^Sc^ inhibitors, with molecular weights of about 300, although their 3-monogallate derivatives were effective, namely **141** and **142**. Noteworthy, compound **143**, embodying a double bond in the central ring with a higher hydroxylation pattern than **139** and **140**, presented inhibitory properties, demonstrating that changing flavan-type structure by a planar highly hydroxylated flavonol analog had important effects on polyphenol inhibitory efficacy. All other compounds **145**–**156**, with a diversity of highly substituted core structures, namely steroid, glycosylated triterpene, aromatics and heteroaromatics, did not show inhibitory activity in the SP-CFC reaction at concentrations up to 100 μM.

**Figure 26:**
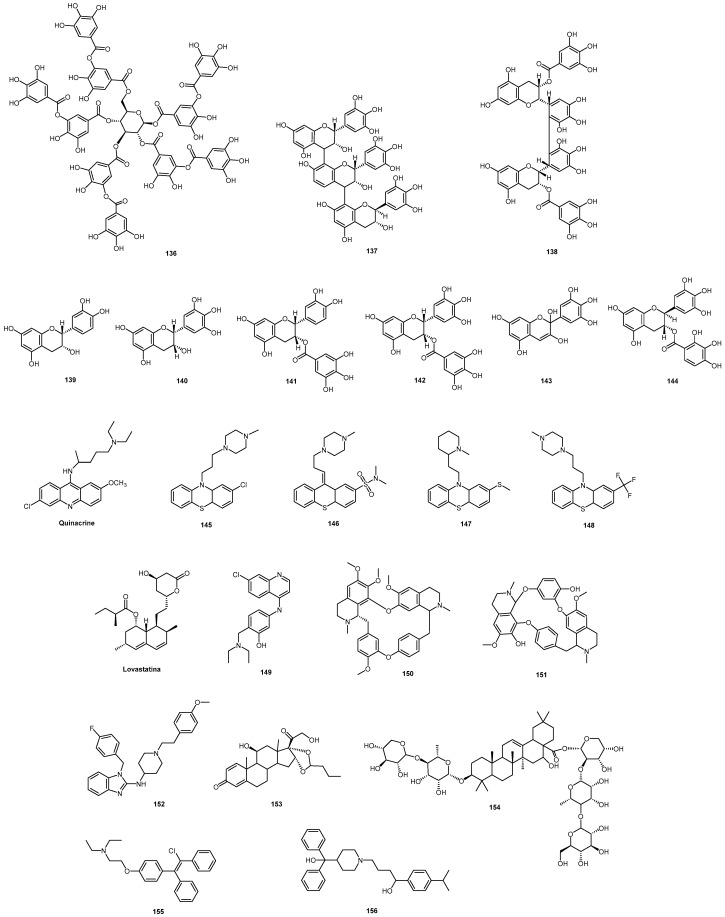
Structure of compounds **136**–**156** studied against both the RML and 22 L scrapie strains [136] and rPrP_90–232_ [139].

In addition, Rambold et al. [139] identified **142** and its diastereoisomer **144** (Figure 26) as potent drugs to interfere with the formation of PrP^Sc^ in ScN2a cells. Noteworthy **141**, with the same configuration as **90** but differing in the hydroxylation pattern, interfered with PrP^Sc^ accumulation efficiently at 200 μM concentration while only 50 μM were needed to have the same effect with **90**. The additional hydroxy group at the trihydroxyphenyl side chain of **142** clearly increases its activity when compared to that of **141**. To test for a direct interaction with PrP, isothermal titration calorimetry (ITC) experiments were carried out using rPrP_90–232_ showing the lack of interaction of **141** with rPrP_90–232_. While **142** had a K_D_ of 130 nM and a ΔH of −43 kJ, no changes in enthalpies with **141** were observed, indicating absence of binding. In addition, compounds **139** and **140** both without the gallate side chain, did not interfere with PrP^Sc^ propagation, endorsing, as reported by Kocisko et al. [136] the gallate side chain as essential for activity.

Some polyphenols may not be the best candidates to cross the blood brain barrier for their water solubility, but these molecular entities may be useful by preventing TSE disease. However, radiolabeled epigallocatechin gallate (EGCG) has been detected in mouse brains after oral administration [140].

**Figure 27:**
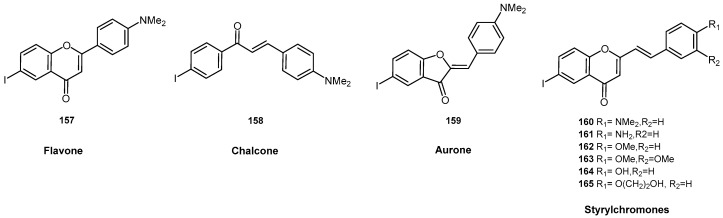
Radioiodinated flavonoids **157**–**159** and styrylchromones **160**–**165** tested by SPECT imaging of PrP^Sc^.

More recently, Fuchigami and co-workers evaluated the potential of radioiodinated flavonoids (flavone **157**, chalcone **158** and aurone **159**) and styryl chromones against PrP^Sc^ (Figure 27) by single photon emission computed tomography (SPECT) imaging of PrP^Sc^ [141].

In vitro binding assays using recombinant mouse PrP (rMoPrP) aggregates revealed that **160** had higher in vitro binding affinity (K_D_ = 24.5 nM and capacity B_max_ = 36.3 pmol/nmol as total density of protein in a sample of tissue) than the three flavonoids (K_d_ = 201 nM and capacity B_max_ = 11.2 pmol/nmol for **157**, K_D_ = 246 nM and capacity B_max_ = 16.7 pmol/nmol for **158**, and K_D_ = 125 nM and capacity B_max_ = 34.9 pmol/nmol for **159**). On the other hand fluorescent imaging using brain sections from mouse-adapted bovine spongiform encephalopathy (mBSE)-infected mice demonstrated that **160** labelled PrP^Sc^-positive prion deposits in the mice brain. Fuchigami et al. were pioneers describing in vivo imaging of PrP^Sc^ deposits in the mBSE-infected mice brain observing high accumulation of the two methoxy derivatives [^125^I]**162** and [^125^I]**163** by in vitro fluorescence and autoradiography experiments. These derivatives showed binding for rMoPrP aggregates with K_i_ = 20.8 and 26.6 nM respectively.

**Scheme 28:**
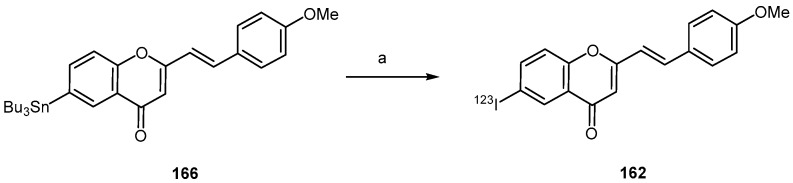
Synthesis of radiolabeled compound **162**. *Reagents and conditions*: [^123^I] NaI, H_2_O_2_, HCl, EtOH, r.t., 10 min.

Radiochemical synthesis of compound **162** was carried out by an iododestannylation reaction of corresponding tributyltin derivative **166** to give compound **162** in 35–44% radiochemical yield and with a radiochemical purity of 98% as depicted in Scheme 28.

**Figure 28:**
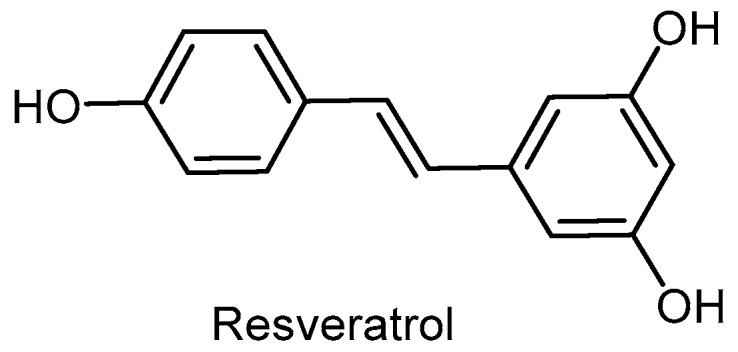
Structure of resveratrol with anti-prion properties.

Other natural polyphenols, structurally based on stilbenes or cinnamic acid derivatives, have shown anti-prion activity, as resveratrol and curcumin. Resveratrol [systematic name: (*E*)-5-(4-hydroxystyryl)benzene-1,3-diol, Figure 28] is produced by grapes, mulberries, and nuts, when attacked by pathogens [142]. Over the past couple of decades, resveratrol has been extensively investigated showing health benefits in neuroprotection, cardioprotection, hepatoprotection, antiinflammation, and cancer prevention [143]. Its neuroprotective properties are not restricted to AD or Parkinson´s diseases being also demonstrated its anti-prion efficacy. Jeong et al. [144] revealed the neuroprotective effects of resveratrol in prion disease demonstrating the prevention of PrP_106–126_-induced neuronal cell death by activating autophagy. They tested the influence of resveratrol treatment on PrP_106–126_-induced apoptosis in neuronal cells (SH-SY5Y cells and SK-N-SH cell lines) by exposing cells to resveratrol with and without PrP_106–126_. Treatment of neuronal cells with PrP_106–126_, without resveratrol, resulted in neurotoxicity, however, resveratrol treatment prevented PrP_106–126_-induced apoptosis in a dose-dependent manner. These results were confirmed by examination of Lactate dehydrogenase assay (LDH) and TUNEL assay [144]. Later on, same authors showed that Sirtuin 1 (Sirt1), a class III histone deacetylase that mediates the protective effects of neurons in neurodegenerative disorders [145], induces autophagy preventing prion peptide neurotoxicity [146].

Wang and co-workers [147] evaluated the treatment of SMB-S15 cells with Sirt1 activators. In the presence of resveratrol, significant reduced levels of PrP^Sc^ were dectected.

To address the anti-prion acitivity of resveratrol, Wang et al. [148] tested the inhibitory activity of resveratrol on prion accumulation in vitro and prion inefectivity in vivo using scrapie-infected cell line SMB-S15. They demonstrated that the amounts of PrP^Sc^ in SMB-S15 cells decreased in a dose-dependent manner in the presence of resveratrol, with EC_50_ = 0.61 μM. The removal of PrP^Sc^ in SMB-S15 cells by resveratrol seemed to be irreversible as no PrP^Sc^ signals could be detected in the resveratrol-treated cells after withdrawal of the drug. Moreover, infectivity of SMB-S15 cells on the sensitive rodent CD1 mice was eradicated after exposing to resveratrol for 1 week.

**Scheme 29:**
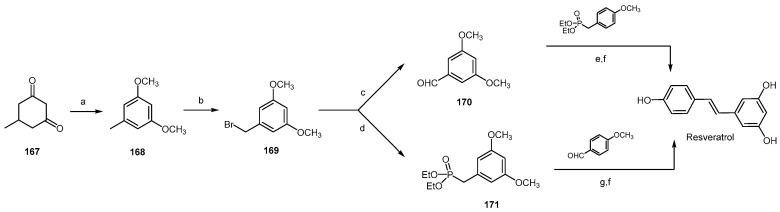
Synthesis of resveratrol. *Reagents and conditions*: (a) MeOH, Pd/C, ethylene, 130 °C, 1 h, 86%, (b) NBS, CH_3_CN, 25 °C, 1 h, 91%, (c) hexamethylenetetramine, H_2_O, reflux, 12 h, 99%, (d) P(OEt)_3_, 80 °C, 12 h, 100%, (e,g) NaH, THF, 28 °C, 3 h, 95% and 94% respectively, (f) AlCl_3_, iPr_2_NH, toluene, 110 °C, 4 h, 85%.

Several synthetic approaches have been described for the synthesis of resveratrol, carried out by Wittig [149] or Horner-Wadsworth-Emmons reactions [150], Perkin condensations [151], and also through metal-catalysed processes, such as cross-metathesis [152] or cross-coupling reactions [153,154]. The most recent synthesis of resveratrol has been carried out by El-Deeb et al. [155] and is shown in Scheme 29. They synthesized resveratrol in five steps with a 62% overall yield. The key step is the oxidation of 5-methylcyclohexane-1,3-dione **167** to 3,5-dimethoxytoluene **168** by hemiacetal formation and subsequent dehydration using ethylene and Pd/C in MeOH at 120 °C (Scheme 29). Then, bromination with NBS in CH_3_CN was carried out to generate compound **169** in 91% yield. Compound **169** was converted in the corresponding aldehyde **170** and the phosphonate **171** to follow two different methodologies as depicted in Scheme 29 for the *trans* double bond formation. They applied the Horner−Wadsworth−Emmons reaction employing on one hand, the reaction of 3,5-dimethoxybenzaldehyde **170** with diethyl 4-methoxybenzylphosphonate and, on the other hand, 4-methoxybenzaldehyde with diethyl 3,5-dimethoxybenzylphosphonate **171**. Both routes gave protected resveratrol in high yield (95 and 96%, respectively) which was later demethylated with the combination of AlCl_3_ and i-Pr_2_NH giving resveratrol in 85% yield (Scheme 29).

**Figure 29:**
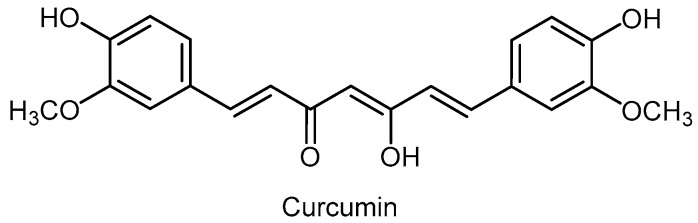
Structure of curcumin with anti-prion properties.

Curcumin [systematic name: 1,7-bis(4-hydroxy-3-methoxyphenyl)hepta-1,6-diene-3,5-dione], (Figure 18) is the main pigment derived *Curcuma longa* that has been identified as an inhibitor of prion fibril formation [156,157] with the ability to cross the blood brain barrier [158]. Curcumin, having an unique conjugated structure including two methoxylated phenols and an enol form of diketone shows a typical radical trapping ability as a chain breaking antioxidant (Figure 29). Structurally, curcumin is similar to Congo red, which is toxic and a poor brain penetrant. However, curcumin does not present apparent toxicity resulting in a promising drug as anti-TSE agent.

Caughey and co-workers have found that curcumin inhibits the accumulation of PrP^Sc^ in scrapie-infected neuroblastoma (scNB) cells [156]. Hafner-Bratkovic et al. showed that this polyphenol binds only to non-native forms of PrP, avoiding prion fibril formation without affecting native PrP [157]. Lin’s lab investigated the anti-amyloidogenic and antioxidant effects of curcumin on the behavior of recombinant murine PrP (mPrP) in a cell-free environment and in murine neuroblastoma (N2a) cells [159]. They evaluated the effect of curcumin on the amyloid formation of mPrP, based on the kinetics of amyloid formation by fluorescence intensity of thioflavin T. ThT-fluorescence increased significantly, but in the presence of curcumin only a weak increase was detected. Analysis of transmission electron microscopy (TEM) images showed mPrP fibrils growing in the absence of curcumin, while in its presence the length of the fibrils was shorter. To investigate the effect of curcumin in cells, they first confirmed the disruption of cell membranes in erythrocytes from mouse blood. Mouse erythrocytes are typically oval and biconcave but when the erythrocytes were co-incubated with mPrP fibrils, cells of wrinkled morphology were formed. However treatment of the erythrocytes with curcumin together with or prior to fibril incubation reduced membrane damage.

Viability of N2a cells turned to increase 33% by treatment with curcumin prior to the co-incubation with amyloid fibrils. An apoptosis assay confirmed that fibril-induced apoptosis was largely weakened when N2a cells were treated with curcumin prior to the fibril treatment. On the other hand, since curcumin is widely applied for its antioxidant properties, it has been investigated its effect on the (ROS) level [159] of N2a cells infected with mPrP fibrils. They found out that mPrP fibril-induced ROS could be completely eliminated by treatment with 2.5 μM of curcumin prior to co-incubation with mPrP fibrils, suggesting that curcumin is involved in the ROS-related signal transduction pathways.

**Scheme 30:**
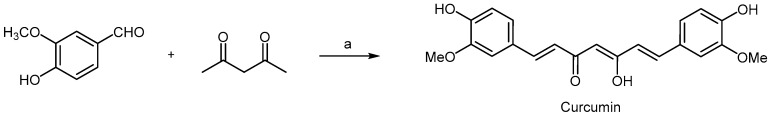
Synthesis of curcumin. *Reagents and conditions*: (a) B_2_O_3_, (BuO)_3_B, *n*-BuNH_2_, AcOEt, r.t., 80% yield.

The major processes of practical utility for the preparation of curcumin follow Pabon’s route [160]. This method involves the reaction of vanillin (4-hydroxybenzaldehyde), acetylacetone and B_2_O_3_ in the presence of tributhyl borate and buthylamine. The reaction is carried out in ethyl acetate at r.t. affording curcumin in 80% yield (Scheme 30).

As shown so far, polyphenols are potent therapeutic agents with a broad range of pharmacological effects, however their biological activity is not completely clear. These compounds modulate many biological pathways and alter functions of different proteins including membrane proteins [161,162,163] pathways. This characteristic of perturbating cell membrane is believed to be the reason for their multiple functions, instead of specific binding to proteins [164] and there is little evidence for direct binding to some of their numerous effector proteins [165].

Amongst the polyphenols mentioned in this section EGCG, resveratrol and curcumin have been described by Ingolfsson et al. [164] to alter bilayer properties and modify membrane protein function. Curcumin displays essentially all known Pan Assay Interference Compounds (PAINS)-type behavior [164,166]. It contains catechol units which are recognizable PAINS motifs interfereing in biological events through different mechanisms [167]. Resveratrol, like many phenols, is readily oxidized and can form reactive quinones [168] which are also PAINS motifs.

The promiscuous activity of these entities should lead us to use them cautiously as control compounds and leave open the challenge to continue structure optimization to investigate their specificity of binding to proteins considered key to the control of neurodegeneration.

## 3. Concluding Remarks

Prion and Alzheimer’s diseases share being protein misfolding disorders. These diseases are fatal, and the latter accounts for most cases of dementia. It is a much more prevalent amyloid disorder than TSEs and, to this point, no effective disease-modifying therapies have been successfully developed. After continuous drug flops in pivotal clinical trials for AD, a new hope has risen since the recent discovery of PrP^C^ as a high-affinity binding partner of amyloid β (Aβ) oligomers, which are the key pathophysiological toxic entities causing neuronal death in AD patients [169]. In fact, post-mortem examinations of brain tissue derived from AD patients have led to the identification of high molecular mass assemblies of Aβ oligomers and PrP^C^ [170]. Moreover, in contrast with AD transgenic mice, PRNP^−/−^ mice with Aβ plaques do not exhibit hippocampal impairment of synaptic plasticity and associated suppression of cognitive function [171]. These data suggest that Aβ oligomer toxicity in AD may in fact be mediated by PrP^C^, and encourage further research focusing on protein-protein interaction inhibitors (PPII) to block the assembly of Aβ-PrP^C^ aggregates in the treatment of AD. By exploring the molecular entities able to intervene with the common mechanisms of prion assembly and prion-promoted neurodegeneration, perhaps in the future new efficacious therapeutic approaches will be achievable and somehow adjustable to all human prions.

## Figures and Tables

**Table 1:** In vitro anti-prion activities of quinacrine and compounds **37**–**39** on murine neuroblastoma cells infected mouse adapted scrapie strains (ScN2a and N167) and a human prion strain (F3), binding responses of test compounds to hPrP_121–231_ by SPR, permeability by PAMPA-BBB assay, log D and efflux ratio across wild type MDCK-MDR1 cell monolayers.

Compound	EC_50_ (μM) ScN2a	EC_50_ (μM) N167	EC_50_ (μM) F3	Binding to hPrP_121–231 (%RUmax)_	Permeability PAMPA-BBB Assay (logD_7.4_)	Efflux Ratio * MDCK-MDR1
Quinacrine	0.23	0.59	1.88	87.3	1.89	5
**37**	0.10	0.42	0.68	120.3	3.36	2.4
**38**	0.42	0.49	0.80	67.5	3.75	-
**39**	0.13	0.23	0.19	169.5	3.41	-

* Ratio of apparent permeability from the basolateral (B) to apical (A) compartment, to apparent permeability from apical (A) to basolateral (B) compartments. Efflux ratio ¼ Papp(B/A)/Papp(A/B).
